# Supplementary material for: Anterior connectivity critical for recovery of connected speech after stroke
Source: Brain Commun. 2022 Oct 19;4(6):fcac266. doi: 10.1093/braincomms/fcac266 (PMC9651028; doi:10.1093/braincomms/fcac266)
Supplement: fcac266_Supplementary_Data [file fcac266_supplementary_data.zip › Original Submission.pdf]

OXFORD UNIVERSITY PRESS | Brain Communications

Anterior Connectivity Critical for Recovery of Connected Speech after Stroke

Journal:	*Brain Communications*
Manuscript ID	BRAINCOM-2022-035
Manuscript Type:	Original Article
Date Submitted by the Author:	29-Jan-2022
Complete List of Authors:	Ding, Junhua; The University of Edinburgh, Psychology<br>schnur, tatiana; Baylor College of Medicine, Neurosurgery
Keywords:	stroke, recovery, connected speech, lesion symptom mapping, white matter tracts, frontal aslant tract

SCHOLARONE™
Manuscripts

Anterior connectivity critical for recovery of connected speech after stroke

Junhua Ding[1] & Tatiana T. Schnur[2]

1. Department of Psychology, University of Edinburgh, UK

2. Departments of Neurosurgery & Neuroscience, Baylor College of Medicine, Houston, Texas

For Review Only

Corresponding author: Tatiana T. Schnur, Dept. of Neurosurgery, Baylor College of Medicine, 1 Baylor Plaza, Houston, TX, 77030, email: ttschnur@gmail.com

Abstract

Connected speech recovers to different degrees across people after left hemisphere stroke, but white matter predictors of differential recovery from the acute stage of stroke are unknown. We assessed changes in lexical-syntactic aspects of connected speech in a longitudinal analysis of 41 individuals (19 females) from the acute stage of LH stroke (within an average 4 days post-stroke) to subacute (within 2 months) and chronic stages (early: 6 months, late: 1 year) while measuring the extent of acute lesions on white-matter tracts to identify tracts predictive of recovery. We found that acute damage to the frontal aslant tract led to decreased recovery of the fluency and structural complexity of connected speech during the year following left hemisphere stroke. In addition, acute damage of the frontal aslant tract and the posterior and long segments of the arcuate fasciculus negatively impacted the recovery of syntactic accuracy, although the associations did not survive correction for multiple comparisons. Results were independent of baseline performance, overall lesion volume, and the proportion damage to tract-adjacent gray matter. This longitudinal analysis from acute to chronic stroke provides the first evidence that recovery of fluent and structurally complex spontaneous connected speech requires intact left frontal connectivity via the frontal aslant tract. That the frontal aslant tract was critical for recovery at early as well as later stages of stroke demonstrates that anterior connectivity plays a lasting and important role for the reorganization of function related to the successful production of connected speech.

Keywords: stroke, recovery, connected speech, lesion symptom mapping, white matter tracts

For Review Only

Introduction

Left-hemisphere stroke is a high-frequency and high-risk disease that profoundly affects communication by impairing the ability to produce words[1,2] and combine them into connected speech.[3–6] To facilitate language intervention, it is important to identify factors which predict degree of connected speech recovery soon after stroke. Damage to disparate brain regions and their connections impairs word retrieval and its recovery[7–9] but the neural predictors of the recovery of connected speech are unknown. In this large-scale longitudinal analysis (n=41), we examined the role of white-matter tract integrity in the recovery of connected speech after acute left hemisphere brain damage during the first year after stroke.

Successfully producing connected speech requires retrieving words and combining them into phrases and sentences while avoiding long hesitations. Connected speech is impaired to different degrees after stroke as a result of damage to disparate brain regions in the left hemisphere. Anteriorly, damage to the left inferior frontal gyrus (IFG) impairs the ability to produce syntactically well-formed sentences and to retrieve grammatically marked words (pars opercularis[5] cf.[10]) and their verbal quantity (i.e. number of total or distinct content words) and quality (i.e. content words/total words) (pars opercularis[3] cf.[11]). Posteriorly, the posterior superior/middle temporal gyri (pSTG/pMTG) and temporoparietal junction (TPJ; angular gyrus and supramarginal gyrus) are also crucial regions for connected speech. Damage negatively impacts sentence complexity and the retrieval of nouns (pSTS and AG[5] cf.[10]). In addition, a reduction in distinct words to total words produced (type/token ratio; pSTG/MTG, AG and SMG[4,6]) and increased semantic and unrelated word errors (pSTG/MTG and AG[12]) are also associated with damage to the temporal pole and anterior portions of all temporal gyri.

For Review Only

Together, a network of anterior to posterior brain regions appears critical for the distinct abilities required to successfully produce connected speech.

The degree to which regions are connected also contributes to connected speech performance and may, at least as seen with general cognitive abilities, better account for impairments in comparison to lesion topography ([13–15] cf.[11]). One well-defined pathway important for connected speech is the arcuate fasciculus (AF). Most studies have focused on the relation between connected speech impairment and damage to the long segment of the AF (LSAF), which connects the posterior part of the pars opercularis of the IFG (BA 44) with middle and inferior temporal gyri.[16,17] Its damage in chronic stroke patients is associated with impairments in different aspects of word retrieval during connected speech including the speed with which overall words and narratively relevant words are produced (fluency[18–21]). Similar impairments in connected speech are also seen after chronic damage to other parts of the AF. For example, damage to the anterior segment of the AF (ASAF), connecting the posterior part of the pars opercularis of the IFG with the supramarginal gyrus is also associated with impairments in the fluency as well as a general composite measure of connected speech (a combination of appropriate information, fluency, syntactic variety and accuracy measures[11,22]). After accounting for the contribution of the ASAF, chronic damage to another tract, the uncinate fasciculus (UF) which connects the IFG (pars orbitalis; BA 47) with the anterior temporal lobe (BAs 20,38[23]) was also associated with speech fluency impairments.[22] Thus, white matter pathways which connect the IFG with other cortical areas play a critical role for lexically-based aspects of connected speech.

For Review Only

However, not all IFG associated tracts are involved in connected speech production. A less-explored tract, the frontal aslant tract (FAT) which connects the pars opercularis of the IFG with the anterior supplementary and pre-supplementary motor areas[24,25] has not been found to impact either lexical or discourse aspects of connected speech.[11,21] Similarly, there is no evidence of which we are aware of a role in connected speech for the IFOF[21,22] which connects the IFG (BAs 44, 45, 47) with the superior ATL and posterior temporal lobe.[26,27] Lastly, with regards to pathways which connect posterior regions, chronic damage to the posterior segment of the AF (PSAF) which connects the pMTG with the angular gyrus in the parietal lobe was not associated with speech fluency of connected speech[22] (cf. [21] for evidence regarding how ventral tract damage to the ILF which connects the ATL (BA 38) with the posterior temporal lobe affects discourse). Thus, the evidence to date suggests that chronic impairments in different aspects of word retrieval during connected speech are associated with damage to tracts which connect the IFG with other regions (LSAF, ASAF, UF) although this relationship does not extend to all anteriorly connected tracts (FAT, IFOF).

It is unclear why damage to white matter tracts which connect different parts of the language network associated with different language functions have not been strongly differentiated with regards to connected speech. However, because study enrollment is usually restricted to those participants with clinically diagnosed aphasia, the severity of language impairments was likely accompanied by more extensive brain damage. As a result, participants may have damage to multiple brain regions simultaneously, thus equally impacting different white matter tracts. Second, some studies used measures combining multiple aspects of connected speech (e.g., WAB-speech fluency[22]) and thus it is unknown which aspect of

For Review Only

connected speech was most strongly related to a particular segment. Lastly, it is possible that white matter tracts become differentiated with regards to language function to different degrees across individuals because function is reorganized by the chronic stroke stage.

In the only study we are aware of reflecting functional reorganization of connected speech after stroke, Keser et al.[21] examined the relationship between the degree of early (within < 3 months after stroke) damage to white matter connectivity and recovery of connected speech in 10 speakers after stroke. No results were significant in the longitudinal analysis after multiple comparison correction. However, before applying correction, the integrity (i.e. radial diffusivity) of the left LSAF within < 3 months after stroke was correlated with lexical-semantic retrieval/informativeness (i.e. the number of content units produced during picture description) during the chronic phase (4-13 months after stroke). This result is similar to results in participants with chronic stroke.[3,18–20] Regarding how early white matter tract damage affects the production of syntactic aspects of connected speech, significant results were obtained only when examining the relationship between early damage and early performance. Damage to the left ILF (i.e. fractional anisotropy) predicted a measure commonly used to examine discourse, that is, the cohesiveness of one sentence to another measured by the use of personal pronouns to refer to previously named agents. However, coherence reflects not only syntax, but also lexical, semantic and executive control abilities[28,29] making it difficult to interpret which aspect of connected speech critically depended on the ILF. These results highlight the potential importance of the AF and ILF for the recovery of broad aspects of connected speech, but the small subject sample size and lack of lexical-syntactic connected

For Review Only

speech measures in participants during the acute stage of stroke leave open the question of which white matter tracts are critical for the recovery of connected speech.

Current Study

Our aim was to predict the recovery of connected speech using the degree to which white matter tracts were damaged within 1-4 days after left hemisphere stroke. To our knowledge there is no evidence to date concerning how white matter tract damage at the acute stage of stroke affects recovery of abilities to combine words into phrases and sentences, both critical components of successfully produced connected speech. Our study offers distinct advances over previous work. First, we used an ecologically relevant measure of connected speech, spontaneous narrative story-telling. Second, we hand-extracted continuous measures of lexical-syntactic connected speech abilities using quantitative production analysis (QPA[30,31]) which reflects more precise measurements of language abilities in comparison to often used qualitative scoring. Third, we examined the integrity of white matter tracts in participants identified with radiological signs of left hemisphere acute stroke independent of language deficit severity which increased variability in lesion size and location[5]. In contrast to speakers with chronic aphasia who typically have large lesions spanning adjacent cortical regions,[32–34] by including participants with smaller lesions we could disentangle the contribution to function of differentially damaged white matter tracts, including tracts which connect gray matter regions previously found to be involved in language production and also additional segments of the AF (i.e. ASAF and PSAF). Fourth, because acute, subacute and chronic phases of stroke display dynamic brain reorganization mechanisms which recruit different brain regions,[35–37] we assessed longitudinal behavior acutely (< 4 days after stroke) and at least one to three

For Review Only

additional time points during the year after stroke (subacute: 2 months; early chronic: 6 months; late chronic: 12 months).

We predicted white-matter tract involvement in recovery of different aspects of connected speech based on previous evidence of the functional necessity of associated gray matter regions. We expected that recovery of fluency and syntactic accuracy would be influenced by the integrity of anterior connectivity between the IFG (pars opercularis) and supplemental motor (FAT), supramarginal gyrus (ASAF) and middle temporal regions (LSAF).[5,11,18–22] We expected the recovery of word retrieval and structural complexity would be influenced by the integrity of posterior connectivity (PSAF, ILF) because these functions are associated with the TPJ or pSTG/pMTG damage.[5] By examining longitudinal recovery in large sample sizes (> 25 participants; total n = 41) at four time points while controlling for baseline performance,[38,39] we tested for specific white-matter mediated mechanisms of recovery from before significant reorganization of brain behavior occurred through subsequent stages of functional reorganization.

For Review Only

Methods

Participants

Forty-one left hemisphere (LH) stroke patients (22 male; 36 right-handed; 1 hemorrhagic stroke; age: M = 61, S.D. = 14, range = 20-85 years; education: M = 14, S.D. = 3, range = 8-23 years) were consecutively recruited and tested during the acute phase of stroke (interval between stroke onset and testing: M = 4 days, S.D. = 2, range = 1-12) and follow-up stages post-stroke (subacute: n = 34, interval: M = 55 days, S.D. = 28, range = 23-124; post 6 months: n = 33; interval: M = 204 days, S.D. = 31, range = 163-294; 12 months: n = 27, interval:

M = 398 days, S.D. = 49, range = 339-510) from the Memorial Hermann, Houston Methodist and St. Luke's hospitals' comprehensive stroke centers in Houston, Texas, USA as part of an ongoing longitudinal project.[5,31,40,41] Subjects met the following inclusion criteria: Native English speaker; No concomitant neurological/psychiatric diseases (e.g., tumor, dementia, epilepsy or depression); no severe visual or auditory deficits. Three patients were recruited with neurological signs of acute LH stroke but no clear lesion was identified from neuroimaging. Three patients had chronic LH lesions (> 15 mm;[42] lesion locations: cerebellum, basal ganglia and medial parietal lobe). To note, we included <10% of patients with prior chronic stroke because our primary aim was to understand the recovery mechanisms after acute stroke damage and with these limited subject inclusions, prior stroke history is not a significant factor accounting for language recovery.[43] The control group consisted of 13 non-brain damaged participants (three male, 11 right-handed) with normal cognitive ability (Mini-Mental State Examination Scores > 26)[44] matched in age and education with the patient group ($|t|$'s < 1.77; p's > 0.08). Mean age and education were 55 (SD = 14, range = 37–78) and 16 (SD = 3; range = 12–22) years, respectively. Informed consent was approved by the Baylor College of Medicine Institutional Review Board.

Connected speech assessment

Participants viewed a picture book of the Cinderella story [45](Jeffers, 2004) for as long as they wished with printed text occluded, and then told the story in their own words without viewing the book.[5,40] Connected speech narratives were transcribed and scored according to the procedures of quantitative production analysis (QPA) [30,46,47] to yield 13 lexical-syntactic measures of connected speech which we z-scored relative to control performance. We used the

For Review Only

principal component analysis (PCA) coefficients generated for four connected speech components from the large acute left hemisphere stroke sample (n = 65) in Ding et al.[5] to calculate four component scores for each participant and then standardized them based on the control cohort for an intuitive interpretation of impairment degree. The four component scores reflected structural complexity, lexical selection, syntactic accuracy and fluency aspects of connected speech. As described in detail by Ding et al., structural complexity reflected the degree of phrase elaboration, number of sentence embeddings, and sentence length. Lexical selection reflected the ability to produce nouns in comparison to verbs, pronouns, and closed-class words. Syntactic accuracy reflected the ability to produce syntactically accurate speech, including more well-formed sentences, more words within as opposed to outside of sentences and increased production of required determiners. Fluency reflected the number of narrative words produced per minute. For the lexical selection component, we multiplied scores by -1 so that lower scores reflect increased impairment. Three patients were removed from the analysis because their acute and/or follow-up z-scores of the syntax component were extreme outliers (< 25% quartile – 3 interquartile range or > 75% + 3 interquartile range). Therefore, the final patient participant sample sizes across the subacute, post 6- and 12- months groups were 32, 31 and 25 participants respectively. We defined the dependent measure of patients' recovery as the difference between component *z*-scores at acute and each follow-up stage.

Neuroimaging acquisition and processing

We acquired diffusion weighted and high-resolution structural scans (T1 and T2 FLAIR) along the axial direction as part of the clinical protocols for admitted acute stroke cases (interval between stroke onset and scan: M = 2 days from stroke onset; S.D. = 2; range = 0-10).

For Review Only

The voxel sizes of diffusion-weighted and structural images were 1*1*4.5 mm, and 0.5 * 0.5 * 4.5 mm, respectively.

Acute lesions were delineated manually. We first co-registered the diffusion weighted images with the high-resolution structural images (T1 or T2) using AFNI (https://afni.nimh.nih.gov/[48]). Lesions were delineated on the diffusion weighted images, using ITK-snap (http://www.itksnap.org/pmwiki/pmwiki.php[49]). Next, we normalized the individual structural images to the Colin-27 template/MNI space using ANTs registration (http://stnava.github.io/ANTs/[50]). Finally, we used the affine parameter and diffeomorphic map from the last step to transform individual masks to the MNI space. Due to MRI contraindication, one patient received a CT scan. This lesion was directly delineated on the Colin 27 template based on the CT image.

To quantify the damage to white matter tracts at the acute stage of stroke, we extracted the intersection volume between acute lesion masks and seven primary tracts. Ventral pathways included the IFOF, ILF and UF and dorsal pathways included the FAT and AF (see Figure 1). With regards to the AF, it was further separated into anterior, long, and posterior segments.[51–53] We defined the tracts using a probabilistic atlas where voxels within white matter tracts were defined using a 50% probability criterion.[25]

For Review Only

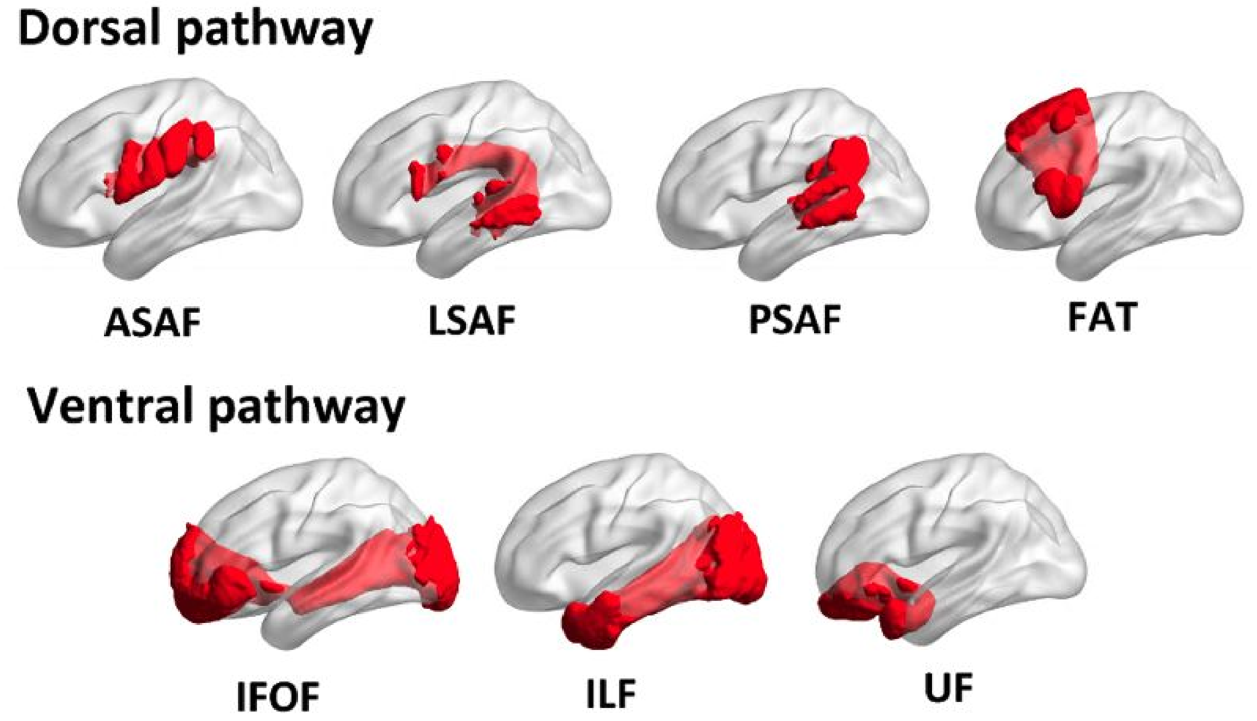

iew Only

Figure 1. The white matter tracts of interest. ASAF: anterior segment of arcuate fasciculus; LSAF: long segment of arcuate fasciculus; PSAF: posterior segment of arcuate fasciculus; FAT: frontal aslant tract; IFOF: inferior fronto-occipital fasciculus; ILF: inferior longitudinal fasciculus; UF: uncinate fasciculus.

Lesion-symptom mapping

To determine the relationship between white matter tracts and connected speech recovery, we conducted a series of lesion-symptom mapping (LSM) analyses. Before executing the LSM analyses, we regressed out participant acute performance (baseline) and total lesion volume from the recovery score.[54,55] We controlled for acute performance because it was highly correlated with degree of recovery (see Results). Tracts were considered damaged if the intersection volume was above 100 mm^3. Then using LESYMAP

(https://dorianps.github.io/LESYMAP/[56]) we conducted a Brunner-Munzel test to compare the recovery scores between tract-preserved and damaged groups where at least >10% of individuals had damage to a given tract.[1,57] To control for multiple comparisons, we applied a Bonferroni correction (corrected $p = 0.05/7$). Lastly, to confirm the specificity of acute white matter tract damage results independent of adjacently located gray matter damage, we replicated results while controlling for the degree of gray matter damage at tract origins and termini. We defined the gray matter damage as the intersection volume between acute lesion masks and gray matter ROIs extracted from the AAL atlas.[58]

Data availability

The data that support the findings of this study are available from the corresponding author, upon reasonable request.

Results

Connected speech recovery from the acute stage of stroke

Fluency of connected speech improved by the subacute timepoint for over 50% of participants in comparison to their impaired baseline acute performance (<-1.67 of controls). However, fluency deficits continued to be a problem at chronic stage timepoints for 41-51% of participants. In contrast, more patients recovered to a neurotypical level in their syntactic ability during connected speech production (80-100% of patients). Regarding lexical ability, 67% of patients recovered to within neurotypical levels across subacute and 6- month time points, while the proportion increased to 80% post 12 months. For structural complexity of connected speech, at the subacute stage, only 67% patients recovered to within neurotypical levels, but the proportion of patients performing within the neurotypical range rose to 80% 6 months post

For Review Only

stroke, and eventually 100% a year after stroke (see Figure 2, Figure 3 and Table 1). In sum, recovery to neurotypical levels of performance for fluency averaged to approximately one in every two patients, while syntactic, lexical selection and structural complexity eventually recovered to neurotypical levels in >80% patients.

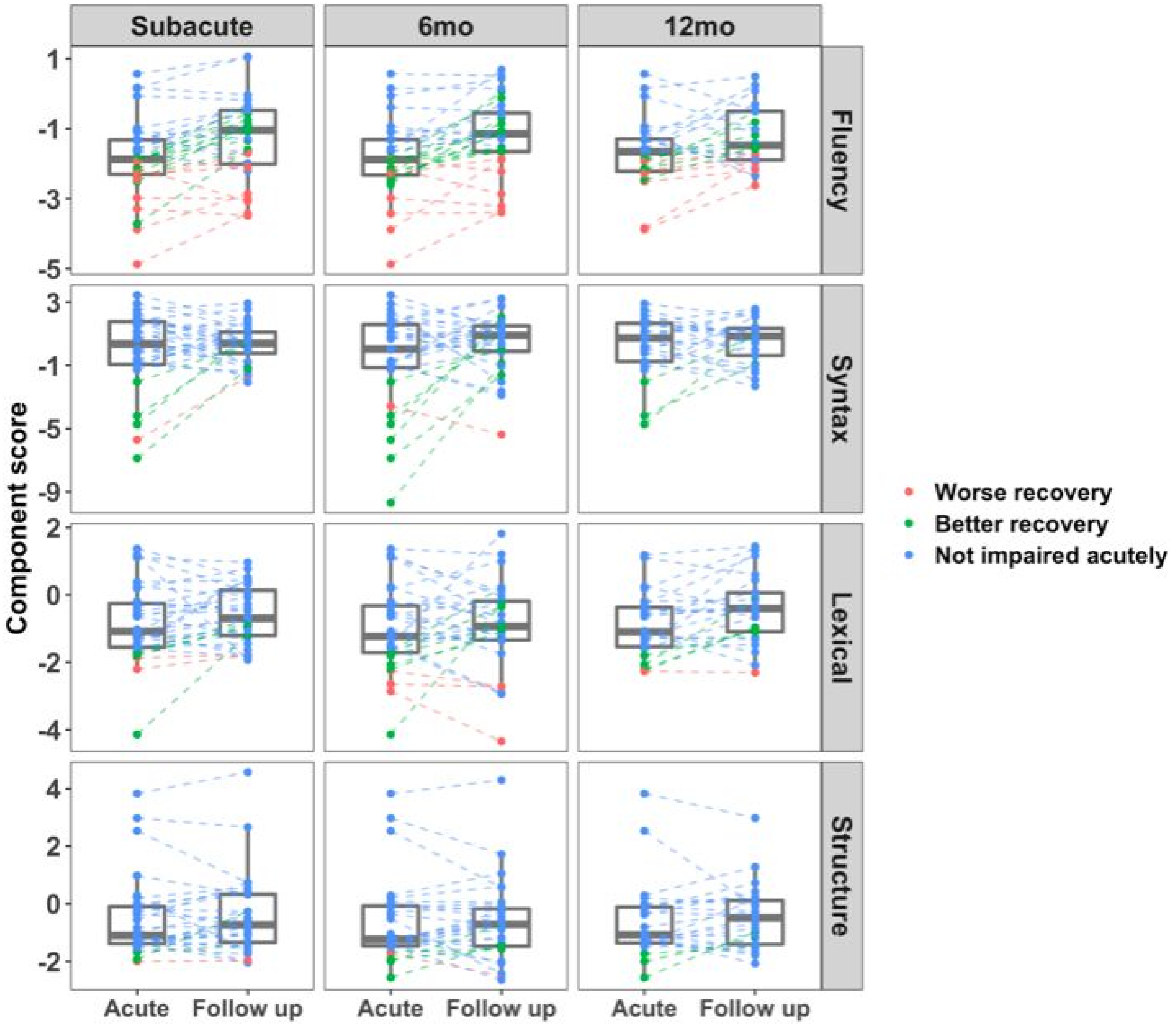

Figure 2. Recovery of four aspects of connected speech from acute to three follow-up time points in the year following stroke. Worse recovery was defined as both acute performance worse than 90% of controls' performance (z's < -1.67) and follow up performance still worse

than 90% of controls (z's < -1.67). In contrast, better recovery was defined as those with impaired acute performance z's < -1.67 but subsequent follow up performance better than 90% of control performance (z's > -1.67). We defined not impaired as individuals who performed better than 90% of controls' performance acutely (z's > -1.67).

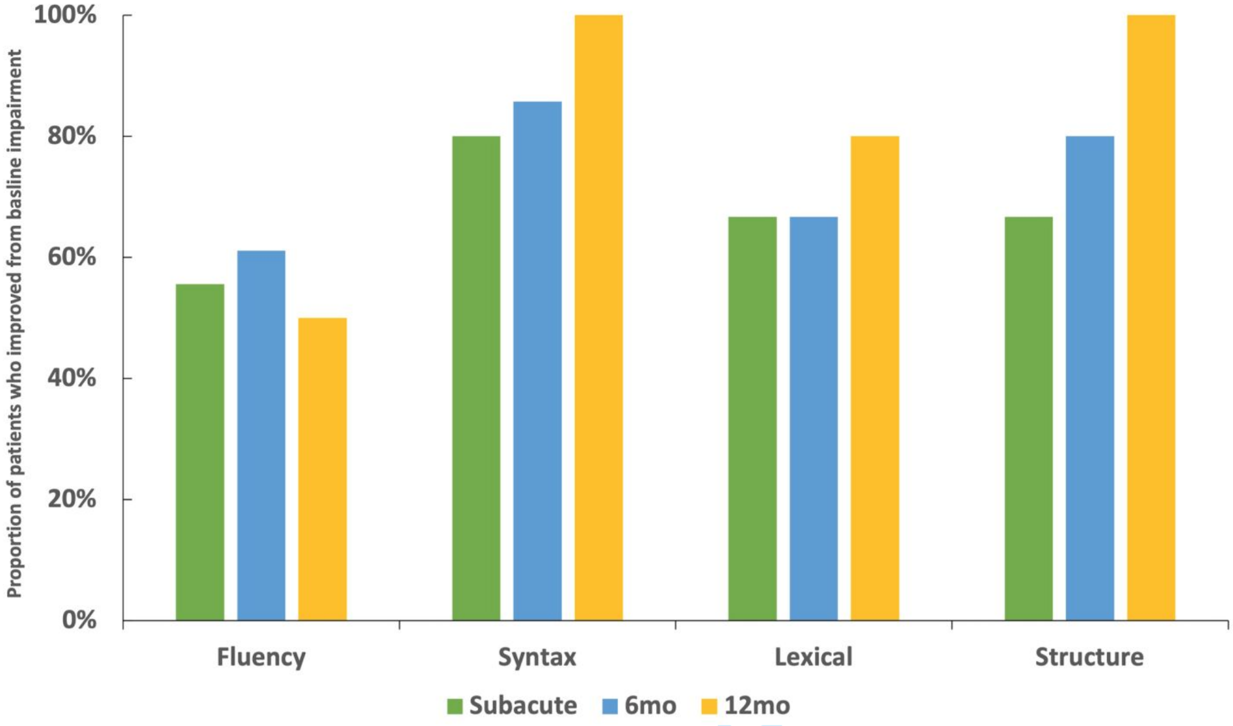

Figure 3. The proportions of patients who recovered to within normal levels (*z* > -1.67) after beginning with impairment acutely (*z* < -1.67) in four aspects of connected speech across three time points in the year following stroke.

Table 1. The number and proportion of patients who recovered to within normal levels (*z* > -1.67) after beginning with impairment acutely (*z* < -1.67) in four aspects of connected speech across three time points in the year following stroke.

	Fluency	Syntax accuracy	Lexical selection	Structural complexity
Subacute (n = 32)	10/18 (56%)	4/5 (80%)	4/6 (67%)	2/3 (67%)
6mo (n = 31)	11/18 (61%)	6/7 (86%)	6/9 (67%)	4/5 (80%)
12mo (n = 25)	6/12 (50%)	3/3 (100%)	4/5 (80%)	3/3 (100%)

Numbers outside parentheses denote the number of patients with impaired ability at the acute stage but within normal range at the follow-up stage (>-1.67) divided by the total number of patients with impaired abilities (<-1.67) at the acute stage. Numbers within parentheses reflect the proportion of patients who improved from acute impairment.

For Review Only

Predicting connected speech recovery from acute white matter tract damage

Figure 4 displays the proportion of patients with acute damage to each white matter tract at each time point. Among the seven tracts we examined, LSAF, FAT and IFOF were damaged in the most patients (n's > 8; 29-44% of patients), while the PSAF and UF had damage to the fewest patients (n's < 7; 16% - 22% of patients).

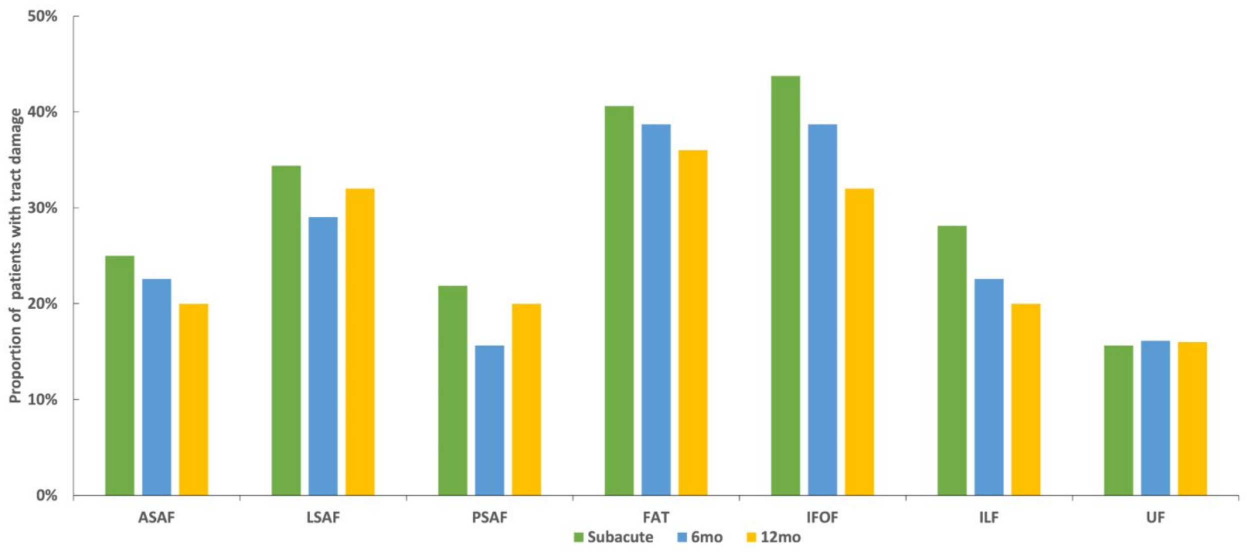

Figure 4. Proportion patients with tract damage for each tract across overlapping patient cohorts at subacute, early chronic (6mo) and late chronic (12mo) time points. ASAF: anterior segment of arcuate fasciculus; LSAF: long segment of arcuate fasciculus; PSAF: posterior segment of arcuate fasciculus; FAT: frontal aslant tract; IFOF: inferior fronto-occipital fasciculus; ILF: inferior longitudinal fasciculus; UF: uncinate fasciculus.

To identify factors potentially confounding relationships between tract damage and connected speech measure recovery, we examined relationships between degree of recovery across time points and demographic variables (age, education, days tested post-stroke), total lesion volume and acute performance (see Supplementary Table 1). The only significant variable associated with degree of connected speech recovery after multiple-comparisons correction (Bonferroni corrected $p < 0.05/5$) was acute baseline performance. Acute performance for all connected speech measures significantly correlated with recovery degree (r values < -0.38; p values < 0.06) except recovery of structural complexity at the subacute stage (r (30) = -0.23, p = 0.20) suggesting that patients with more severe impairments had more room

For Review Only

for improvement. As a result, we included acute performance as a confounding variable in subsequent analyses.

Figure 5, Supplementary Figure 1 and Supplemental Table 2 show the LSM results using white matter tract acute damage to predict connected speech recovery controlling for acute baseline performance and lesion volume. During the subacute stage after stroke, acute FAT damage led to decreased recovery of fluency (z = 2.79, p = 0.003) and at the chronic stages (six – 12 months post-stroke) significantly decreased recovery of structural complexity (z values > 2.53, p values < 0.006) (see Figure 4). Several other findings were significant but did not survive correction for multiple comparisons (see Supplementary Figure 1). At the subacute stage, acute FAT damage predicted decreased recovery of syntactic abilities (z = 1.90, p = 0.03). Chronically, decreased recovery of syntax 6 months (z =2.29, p = 0.01) and 12 months post stroke (z = 2.28, p = 0.01) was associated with acute damage to long and posterior segments of the AF. We replicated these findings when additionally controlling for the degree of gray matter damage at the origins and termini for each tract (p's < .03 save for the marginally significant relationship between acute PSAF damage and degree of syntactic complexity recovery at 6 months post-stroke, p = .08; see Supplementary Table 3). In sum, acute damage to the FAT, and to a less consistent degree the AF contributed to decreased recovery of connected speech abilities at different times points after stroke.

For Review Only

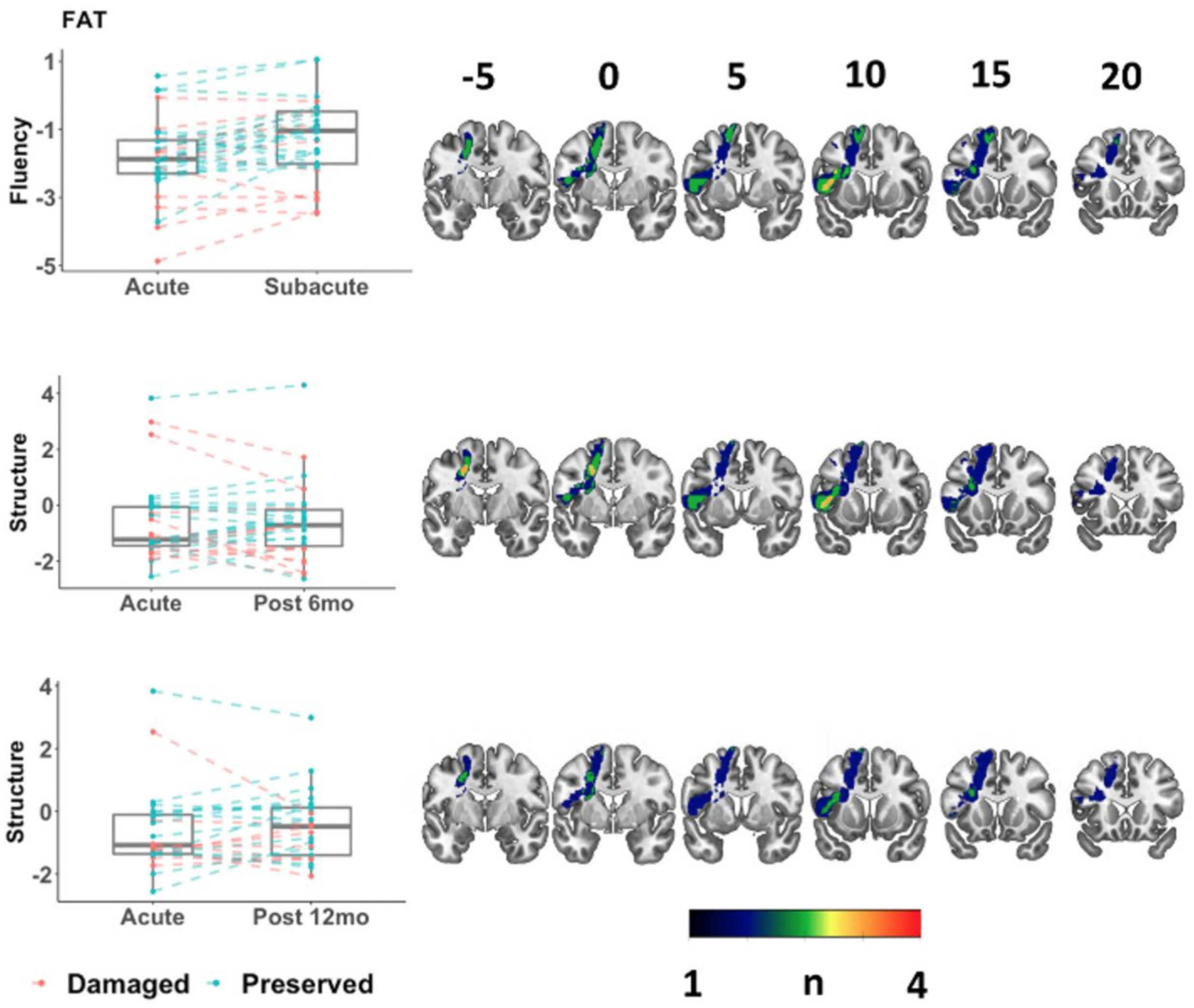

Figure 5. The white matter tracts whose acute damage predicted decreased recovery in four aspects of connected speech from the acute stage to three time points in the year following stroke (subacute, 6mo, and 12mo post stroke) after multiple-comparison correction ($p < 0.05/7$). The box plots show the acute and follow-up connected speech scores clustered by tract damage. Red lines indicate subjects with damaged tracts, while green lines indicate subjects with spared tracts. The brain maps show lesion overlaps of the damaged group on the corresponding tracts. The numbers above the brain slices indicate MNI y coordinates.

Discussion

We report the first large-scale longitudinal study of the impact of acute white matter damage on recovery of connected speech from the acute to chronic stages of left hemisphere stroke. The most frequent impairments in connected speech during the first year of recovery in those able to produce measurable connected speech acutely were reduced words per minute (~25% of the cohort) followed by reduced ability to produce nouns in comparison to other types of words, decreased syntactic accuracy and reduced structural complexity (< ~10%). White matter damage measured within the first week of stroke to a left dorsal pathway, the frontal aslant tract predicted worse subacute recovery of fluency and decreased chronic recovery of the structural complexity of connected speech. Decreased recovery of syntactic accuracy was associated with acute damage to tracts within the dorsal pathway, the frontal aslant tract and the long and posterior segments of the arcuate fasciculus, although these associations did not survive correction for multiple comparisons. Acute white matter integrity was a better predictor of recovery of connected speech than overall lesion volume, damage to tract adjacent gray matter regions, or baseline acute connected speech performance. Our results provide novel evidence that recovery of connected speech after stroke depends on acute connectivity within frontal cortical regions and to a lesser degree between frontal-temporal regions.

Connected speech impairments and recovery

Different aspects of connected speech production improved following the acute stage of stroke. To quantify connected speech, we used Ding et al.'s[5] principal component analysis (PCA) coefficients generated for four connected speech components to calculate component scores

For Review Only

for each participant. Of those who returned subacutely following stroke, reduced production of words per minute, i.e. fluency affected 56% of these subjects acutely. Approximately half of the impaired cohort recovered to within neurotypical fluency performance by the subacute stage of stroke. However, the remaining proportion of subjects with fluency impairments did not markedly change over the next year, suggesting a long-lasting deficit.

A second impairment in connected speech was reduced ability to produce nouns in comparison to other types of words. At the acute stage, 28% of participants demonstrated lexical selection impairments. Slightly more than half (67%) recovered to neurotypical levels by the subacute and early chronic stages. By a year post-stroke, almost all participants (80%) recovered to within the neurotypical range.

Recovery was better for other aspects of connected speech. The ability to produce syntactically accurate speech, including more well-formed sentences, more words within as opposed to outside of sentences and increased production of required determiners was impaired acutely in an average of 18% of participants but by the late chronic stage all subjects recovered to within a neurotypical range of performance. Similarly of 13% who suffered acute impairment producing structurally complex speech including elaborated phrases, sentence embeddings, and longer sentences, all participants recovered by the late chronic stage.

Overall, of participants with acute connected speech impairments, connected speech improved in over 50% of returning participants by the subacute stage of stroke. Encouragingly, nearly all participants improved their connected speech by the late chronic stage of stroke, except for fluency, which remained impaired in almost half the cohort. It is important to note that we enrolled patients who could produce measurable connected speech independent of

For Review Only

any clinical diagnosis of language impairment. Thus, it is striking that even when not including patients with severe production deficits, only half of those with impaired fluency recovered. In comparison, more than 80% of participants by the late chronic stage produced syntactically accurate, structurally complex, and lexically diverse connected speech. However, it remains an open question the degree to which more severe connected speech deficits follow the connected speech patterns of improvement observed here.

Acute white matter tract damage and connected speech recovery

We examined how damage to white matter tracts at the acute phase of stroke impacted the recovery of connected speech during reorganization of function across the subacute, early chronic and late chronic phases of stroke. Of several dorsal and ventral white matter tracts we examined across different stages of recovery, the frontal aslant tract (FAT) was the only tract significantly related with connected speech recovery after multiple comparison correction. At the subacute stage of stroke, acute FAT damage decreased degree of fluency recovery as expected. That acute FAT damage reduces early recovery of fluency during connected speech is consistent with evidence in chronic aphasia demonstrating that FAT integrity is critical for fluency as measured by words per minute during connected speech [3](cf. [59] for similar results in primary progressive aphasia), qualitative assessment of fluency [60] and word generation during categorical fluency tasks.[61,62] In contrast, we did not find that acute damage to tracts connecting frontal with posterior regions including the anterior segment of the arcuate fasciculus or a ventral tract, the uncinate fasciculus predicted fluency impairments as has been demonstrated in chronic aphasia.[22,60] These differences may be due to previous studies' measurement of fluency with a qualitative 10-pt scale assessment (via the Western Aphasia

For Review Only

Battery[63]) which captures other functions including prosody, syntax, and lexical retrieval.[60] At the early and late chronic stages of stroke, acute FAT damage also decreased degree of structural complexity recovery. These results were inconsistent with our predictions as only damage to left temporal-parietal regions distant from the FAT has been associated with impairments in the structural complexity of speech, both acutely[5] and chronically.[10,64–66] Thus, this latter result suggests that cortical regions associated with the frontal aslant tract, like the IFG, may functionally reorganize to support posterior temporo-parietal processes involved in creating more structurally complex connected speech in chronic stage. Together, these results demonstrate the critical role of frontal connectivity for connected speech during reorganization of function.

Regarding the significant associations that did not survive statistical correction for multiple comparisons, recovery of syntactic accuracy during connected speech was associated with both the frontal aslant tract (FAT) and several segments of an additional dorsal tract, the arcuate fasciculus (AF). Specifically, acute damage to the FAT impacted recovery of syntactic accuracy subacutely, while acute damage to the long and posterior segments of the AF predicted worse syntactic accuracy recovery at late (12 months post) and early (6 months post) stages of chronic stroke, respectively. The FAT and long segment AF results were consistent with our predictions as both the FAT and the long segment of AF are associated with the inferior frontal gyrus, connecting it either with the pre-supplemental/anterior motor areas (FAT) or middle temporal gyrus (long segment of AF[16]). To our knowledge there are no longitudinal studies of white matter tract damage and recovery of syntactic accuracy in connected speech. Work using voxel-wise correlation analyses between gray matter damage

For Review Only

and behavior suggests a role in syntactic accuracy for cortical regions which the FAT connects. For example, damage to the anterior aspect of the IFG reduced syntactic accuracy during production in acute stroke[5] and has been associated with qualitative assessments of agrammatic speech in chronic stroke.[10] In chronic stroke, the long segment of AF is necessary for syntactic processing for sentence production[67] and comprehension[68] supporting the association we observed at the late chronic stage between the long segment of AF and degree of syntax recovery. In contrast, the posterior AF segment is not associated with frontal cortical regions as it connects the middle temporal gyrus with the angular gyrus.[16] Although we did not predict the posterior AF's role in syntactic recovery, there is some evidence at the chronic stage of stroke that the posterior AF segment is critical for processing syntax during sentence comprehension,[69] which suggests a potential additional mechanism by which syntax for production can be recovered during the early stages of reorganization. Understanding the exact dynamics of how the frontal aslant tract and the different segments of the AF interact over time to support recovery of syntactic accuracy during production will be a fruitful direction for future studies. The message we take away from these results is that at a minimum, reorganization of syntactic function for production depends on connections and regions firmly placed within dorsal pathways and brain regions.

Other predictors of connected speech recovery

Lastly, it is important to note that lesion volume did not predict the degree of connected speech recovery at any time point in the year after stroke. Although lesion volume often predicts language deficit severity in stroke (e.g.[6,19,34,36,67,70,71]; cf.[18]), lesion volume does not predict the degree of general language[72] or domain-specific cognitive function[73] recovery.

For Review Only

Instead, initial performance was more important to the recovery degree (cf.[72,73]). We also found acute performance a significant diagnostic of connected speech recovery outcomes which is consistent with longitudinal recovery patterns of upper-extremity movement after stroke.[74]

Limitations

There are several inherent limitations which arise as a result of the difficulty in conducting large-scale longitudinal analyses of recovery from stroke. First, even though participants significantly overlapped across the subacute, early and late chronic timepoints (between 66-88% of participants), for a variety of reasons not every patient was tested at all follow-up time points. To maximize power to detect factors which affected recovery, we included the largest number of subjects tested both acutely and at a single subsequent time point. Although this approach helped avoid type II statistical errors, i.e. false negatives, it rendered direct comparisons between time points difficult to interpret. Second, in our cohort, there was a low prevalence of participants with severe impairments in the syntactic accuracy or structural complexity of their connected speech. We conjecture that patients with these types of severe connected speech impairments could not produce measurable spontaneous speech acutely, an exclusion criteria for this study. To understand whether different recovery mechanisms occur in those with more severe connected speech deficits, our laboratory is engaged in an ongoing effort to expand subject recruitment to these clinical populations. Finally, future work will test the degree to which the right hemisphere contributes to recovery of connected speech after stroke as evidence suggests its role in language recovery (cf.[20,70,75]).

For Review Only

Conclusion

This longitudinal analysis from acute to chronic stroke provides the first evidence that recovery of fluent and structurally complex spontaneous connected speech requires intact left frontal connectivity via the frontal aslant tract. That the frontal aslant tract was critical for recovery at early as well as later stages of stroke demonstrates it plays a lasting and important role for the reorganization of function related to the successful production of connected speech. These results suggest that measures of acute cortical disconnection may be useful biomarkers to identify patients who will most benefit from early interventions to remediate chronic connected speech impairments.

For Review Only

References

1. Mirman D, Chen Q, Zhang Y, et al. Neural organization of spoken language revealed by lesion–symptom mapping. *Nat Commun*. 2015;6(1):6762. doi:10.1038/ncomms7762

2. Schwartz MF, Dell GS, Martin N, Gahl S, Sobel P. A case-series test of the interactive two-step model of lexical access: Evidence from picture naming. *J Mem Lang*. 2006;54(2):228-264. doi:10.1016/j.jml.2005.10.001

3. Alyahya RSW, Halai AD, Conroy P, Lambon Ralph MA. A unified model of post-stroke language deficits including discourse production and their neural correlates. *Brain*. 2020;143(5):1541-1554. doi:10.1093/brain/awaa074

4. Borovsky A, Saygin AP, Bates E, Dronkers N. Lesion correlates of conversational speech production deficits. *Neuropsychologia*. 2007;45(11):2525-2533. doi:10.1016/j.neuropsychologia.2007.03.023

5. Ding J, Martin RC, Hamilton AC, Schnur TT. Dissociation between frontal and temporal-parietal contributions to connected speech in acute stroke. *Brain*. 2020;143(3):862-876. doi:10.1093/brain/awaa027

6. Halai AD, Woollams AM, Lambon Ralph MA. Using principal component analysis to capture individual differences within a unified neuropsychological model of chronic post-stroke aphasia: Revealing the unique neural correlates of speech fluency, phonology and semantics. *Cortex*. 2017;86:275-289. doi:10.1016/j.cortex.2016.04.016

7. Hillis AE, Beh YY, Sebastian R, et al. Predicting recovery in acute poststroke aphasia. *Ann Neurol*. 2018;83(3):612-622. doi:10.1002/ana.25184

8. Meier EL, Johnson JP, Pan Y, Kiran S. The utility of lesion classification in predicting language and treatment outcomes in chronic stroke-induced aphasia. *Brain Imaging Behav*. 2019;13(6):1510-1525. doi:10.1007/s11682-019-00118-3

9. van Hees S, McMahon K, Angwin A, de Zubicaray G, Read S, Copland DA. Changes in White Matter Connectivity Following Therapy for Anomia Post stroke. *Neurorehabil Neural Repair*. 2014;28(4):325-334. doi:10.1177/1545968313508654

10. Matchin W, Basilakos A, Stark BC, den Ouden DB, Fridriksson J, Hickok G. Agrammatism and Paragrammatism: A Cortical Double Dissociation Revealed by Lesion-Symptom Mapping. *Neurobiol Lang*. 2020;1(2):208-225. doi:10.1162/nol_a_00010

11. Gajardo-Vidal A, Lorca-Puls DL, team P, et al. Damage to Broca's area does not contribute to long-term speech production outcome after stroke. *Brain*. 2021;144(3):817-832. doi:10.1093/brain/awaa460

For Review Only

12. Stark BC, Basilakos A, Hickok G, Rorden C, Bonilha L, Fridriksson J. Neural organization of speech production: A lesion-based study of error patterns in connected speech. *Cortex*. 2019;117:228-246. doi:10.1016/j.cortex.2019.02.029

13. Siegel JS, Ramsey LE, Snyder AZ, et al. Disruptions of network connectivity predict impairment in multiple behavioral domains after stroke. *Proc Natl Acad Sci*. 2016;113(30):E4367-E4376. doi:10.1073/pnas.1521083113

14. Thiebaut de Schotten M, Foulon C, Nachev P. Brain disconnections link structural connectivity with function and behaviour. *Nat Commun*. 2020;11(1):5094. doi:10.1038/s41467-020-18920-9

15. Reber J, Hwang K, Bowren M, et al. Cognitive impairment after focal brain lesions is better predicted by damage to structural than functional network hubs. *Proc Natl Acad Sci*. 2021;118(19). doi:10.1073/pnas.2018784118

16. Catani M, Jones DK, Ffytche DH. Perisylvian language networks of the human brain. *Ann Neurol*. 2005;57(1):8-16. doi:10.1002/ana.20319

17. Bernard F, Zemmoura I, Ter Minassian A, Lemée JM, Menei P. Anatomical variability of the arcuate fasciculus: a systematical review. *Surg Radiol Anat*. 2019;41(8):889-900. doi:10.1007/s00276-019-02244-5

18. Marchina S, Zhu LL, Norton A, Zipse L, Wan CY, Schlaug G. Impairment of Speech Production Predicted by Lesion Load of the Left Arcuate Fasciculus. *Stroke*. 2011;42(8):2251-2256. doi:10.1161/STROKEAHA.110.606103

19. Wang J, Marchina S, Norton A, Wan C, Schlaug G. Predicting speech fluency and naming abilities in aphasic patients. *Front Hum Neurosci*. 2013;7. Accessed January 19, 2022. https://www.frontiersin.org/article/10.3389/fnhum.2013.00831

20. Pani E, Zheng X, Wang J, Norton A, Schlaug G. Right hemisphere structures predict poststroke speech fluency. *Neurology*. 2016;86(17):1574-1581. doi:10.1212/WNL.0000000000002613

21. Keser Z, Meier EL, Stockbridge MD, Hillis AE. The role of microstructural integrity of major language pathways in narrative speech in the first year after stroke. *J Stroke Cerebrovasc Dis*. 2020;29(9):105078. doi:10.1016/j.jstrokecerebrovasdis.2020.105078

22. Fridriksson J, Guo D, Fillmore P, Holland A, Rorden C. Damage to the anterior arcuate fasciculus predicts non-fluent speech production in aphasia. *Brain*. 2013;136(11):3451-3460. doi:10.1093/brain/awt267

23. Von Der Heide RJ, Skipper LM, Klobusicky E, Olson IR. Dissecting the uncinate fasciculus: disorders, controversies and a hypothesis. *Brain*. 2013;136(6):1692-1707. doi:10.1093/brain/awt094

For Review Only

24. Catani M, Dell'Acqua F, Vergani F, et al. Short frontal lobe connections of the human brain. *Cortex*. 2012;48(2):273-291. doi:10.1016/j.cortex.2011.12.001

25. Rojkova K, Volle E, Urbanski M, Humbert F, Dell'Acqua F, Thiebaut de Schotten M. Atlasing the frontal lobe connections and their variability due to age and education: a spherical deconvolution tractography study. *Brain Struct Funct*. 2016;221(3):1751-1766. doi:10.1007/s00429-015-1001-3

26. Martino J, Brogna C, Robles SG, Vergani F, Duffau H. Anatomic dissection of the inferior fronto-occipital fasciculus revisited in the lights of brain stimulation data. *Cortex*. 2010;46(5):691-699. doi:10.1016/j.cortex.2009.07.015

27. Wu Y, Sun D, Wang Y, Wang Y. Subcomponents and Connectivity of the Inferior Fronto-Occipital Fasciculus Revealed by Diffusion Spectrum Imaging Fiber Tracking. *Front Neuroanat*. 2016;10. Accessed January 19, 2022. https://www.frontiersin.org/article/10.3389/fnana.2016.00088

28. Stockbridge MD, Berube S, Goldberg E, et al. Differences in linguistic cohesion within the first year following right- and left-hemisphere lesions. *Aphasiology*. 2021;35(3):357-371. doi:10.1080/02687038.2019.1693026

29. Hoffman P, Cogdell-Brooke L, Thompson HE. Going off the rails: Impaired coherence in the speech of patients with semantic control deficits. *Neuropsychologia*. 2020;146:107516. doi:10.1016/j.neuropsychologia.2020.107516

30. Rochon E, Saffran EM, Berndt RS, Schwartz MF. Quantitative Analysis of Aphasic Sentence Production: Further Development and New Data. *Brain Lang*. 2000;72(3):193-218. doi:10.1006/brln.1999.2285

31. Fromm D, Katta S, Paccione M, et al. A Comparison of Manual Versus Automated Quantitative Production Analysis of Connected Speech. *J Speech Lang Hear Res*. 2021;64(4):1271-1282. doi:10.1044/2020_JSLHR-20-00561

32. Ochfeld E, Newhart M, Molitoris J, et al. Ischemia in Broca Area Is Associated With Broca Aphasia More Reliably in Acute Than in Chronic Stroke. *Stroke*. 2010;41(2):325-330. doi:10.1161/STROKEAHA.109.570374

33. Shahid H, Sebastian R, Schnur TT, et al. Important considerations in lesion-symptom mapping: Illustrations from studies of word comprehension. *Hum Brain Mapp*. 2017;38(6):2990-3000. doi:10.1002/hbm.23567

34. Yourganov G, Fridriksson J, Rorden C, Gleichgerrcht E, Bonilha L. Multivariate Connectome-Based Symptom Mapping in Post-Stroke Patients: Networks Supporting Language and Speech. *J Neurosci*. 2016;36(25):6668-6679. doi:10.1523/JNEUROSCI.4396-15.2016

For Review Only

35. Saur D, Lange R, Baumgaertner A, et al. Dynamics of language reorganization after stroke. *Brain*. 2006;129(6):1371-1384. doi:10.1093/brain/awl090

36. Stockert A, Wawrzyniak M, Klingbeil J, et al. Dynamics of language reorganization after left temporo-parietal and frontal stroke. *Brain*. 2020;143(3):844-861. doi:10.1093/brain/awaa023

37. Stefaniak JD, Halai AD, Lambon Ralph MA. The neural and neurocomputational bases of recovery from post-stroke aphasia. *Nat Rev Neurol*. 2020;16(1):43-55. doi:10.1038/s41582-019-0282-1

38. Lazar RM, Minzer B, Antoniello D, Festa JR, Krakauer JW, Marshall RS. Improvement in Aphasia Scores After Stroke Is Well Predicted by Initial Severity. *Stroke*. 2010;41(7):1485-1488. doi:10.1161/STROKEAHA.109.577338

39. Saur D, Ronneberger O, Kümmerer D, Mader I, Weiller C, Klöppel S. Early functional magnetic resonance imaging activations predict language outcome after stroke. *Brain*. 2010;133(4):1252-1264. doi:10.1093/brain/awq021

40. Martin RC, Schnur TT. Independent contributions of semantic and phonological working memory to spontaneous speech in acute stroke. *Cortex*. 2019;112:58-68. doi:10.1016/j.cortex.2018.11.017

41. Martin RC, Ding J, Hamilton AC, Schnur TT. Working Memory Capacities Neurally Dissociate: Evidence from Acute Stroke. *Cereb Cortex Commun*. 2021;2(2):tgab005. doi:10.1093/texcom/tgab005

42. Corbetta M, Ramsey L, Callejas A, et al. Common Behavioral Clusters and Subcortical Anatomy in Stroke. *Neuron*. 2015;85(5):927-941. doi:10.1016/j.neuron.2015.02.027

43. Meier EL, Sheppard SM, Goldberg EB, et al. Naming errors and dysfunctional tissue metrics predict language recovery after acute left hemisphere stroke. *Neuropsychologia*. 2020;148:107651. doi:10.1016/j.neuropsychologia.2020.107651

44. Folstein MF, Robins LN, Helzer JE. The Mini-Mental State Examination. *Arch Gen Psychiatry*. 1983;40(7):812. doi:10.1001/archpsyc.1983.01790060110016

45. Ehrlich A, Perrault C, Jeffers S. *Cinderella*. Dutton Children's Books Children's; 2004.

46. Gordon JK. A quantitative production analysis of picture description. *Aphasiology*. 2006;20(2-4):188-204. doi:10.1080/02687030500472777

47. Saffran EM, Berndt RS, Schwartz MF. The quantitative analysis of agrammatic production: Procedure and data. *Brain Lang*. 1989;37(3):440-479. doi:10.1016/0093-934X(89)90030-8

For Review Only

48. Cox RW. AFNI: Software for Analysis and Visualization of Functional Magnetic Resonance Neuroimages. *Comput Biomed Res*. 1996;29(3):162-173. doi:10.1006/cbmr.1996.0014

49. Yushkevich PA, Piven J, Hazlett HC, et al. User-guided 3D active contour segmentation of anatomical structures: Significantly improved efficiency and reliability. *NeuroImage*. 2006;31(3):1116-1128. doi:10.1016/j.neuroimage.2006.01.015

50. Avants BB, Epstein CL, Grossman M, Gee JC. Symmetric diffeomorphic image registration with cross-correlation: Evaluating automated labeling of elderly and neurodegenerative brain. *Med Image Anal*. 2008;12(1):26-41. doi:10.1016/j.media.2007.06.004

51. Catani M, Allin MPG, Husain M, et al. Symmetries in human brain language pathways correlate with verbal recall. *Proc Natl Acad Sci*. 2007;104(43):17163-17168. doi:10.1073/pnas.0702116104

52. Forkel SJ, Rogalski E, Sancho ND, et al. Anatomical evidence of an indirect pathway for word repetition. *Neurology*. 2020;94(6):e594-e606. doi:10.1212/WNL.0000000000008746

53. López-Barroso D, Catani M, Ripollés P, Dell'Acqua F, Rodríguez-Fornells A, Diego-Balaguer R de. Word learning is mediated by the left arcuate fasciculus. *Proc Natl Acad Sci*. 2013;110(32):13168-13173. doi:10.1073/pnas.1301696110

54. DeMarco AT, Turkeltaub PE. A multivariate lesion symptom mapping toolbox and examination of lesion-volume biases and correction methods in lesion-symptom mapping. *Hum Brain Mapp*. 2018;39(11):4169-4182. doi:10.1002/hbm.24289

55. Sperber C, Karnath HO. On the validity of lesion-behaviour mapping methods. *Neuropsychologia*. 2018;115:17-24. doi:10.1016/j.neuropsychologia.2017.07.035

56. Pustina D, Avants B, Faseyitan OK, Medaglia JD, Coslett HB. Improved accuracy of lesion to symptom mapping with multivariate sparse canonical correlations. *Neuropsychologia*. 2018;115:154-166. doi:10.1016/j.neuropsychologia.2017.08.027

57. Lacey EH, Skipper-Kallal LM, Xing S, Fama ME, Turkeltaub PE. Mapping Common Aphasia Assessments to Underlying Cognitive Processes and Their Neural Substrates. *Neurorehabil Neural Repair*. 2017;31(5):442-450. doi:10.1177/1545968316688797

58. Tzourio-Mazoyer N, Landeau B, Papathanassiou D, et al. Automated Anatomical Labeling of Activations in SPM Using a Macroscopic Anatomical Parcellation of the MNI MRI Single-Subject Brain. *NeuroImage*. 2002;15(1):273-289. doi:10.1006/nimg.2001.0978

59. Catani M, Mesulam MM, Jakobsen E, et al. A novel frontal pathway underlies verbal fluency in primary progressive aphasia. *Brain*. 2013;136(8):2619-2628. doi:10.1093/brain/awt163

For Review Only

60. Basilakos A, Fillmore PT, Rorden C, Guo D, Bonilha L, Fridriksson J. Regional White Matter Damage Predicts Speech Fluency in Chronic Post-Stroke Aphasia. *Front Hum Neurosci*. 2014;8. Accessed January 19, 2022. https://www.frontiersin.org/article/10.3389/fnhum.2014.00845

61. Li M, Zhang Y, Song L, et al. Structural connectivity subserving verbal fluency revealed by lesion-behavior mapping in stroke patients. *Neuropsychologia*. 2017;101:85-96. doi:10.1016/j.neuropsychologia.2017.05.008

62. Foulon C, Cerliani L, Kinkingnéhun S, et al. Advanced lesion symptom mapping analyses and implementation as BCBtoolkit. *GigaScience*. 2018;7(3):giy004. doi:10.1093/gigascience/giy004

63. Kertesz A. Western Aphasia Battery--Revised. Published online 2006. doi:10.1037/t15168-000

64. den Ouden DB, Malyutina S, Basilakos A, et al. Cortical and structural-connectivity damage correlated with impaired syntactic processing in aphasia. *Hum Brain Mapp*. 2019;40(7):2153-2173. doi:10.1002/hbm.24514

65. Lukic S, Bonakdarpour B, Ouden DD, Price C, Thompson C. Neural Mechanisms of Verb and Sentence Production: A Lesion-deficit Study. *Procedia - Soc Behav Sci*. 2013;94:34-35. doi:10.1016/j.sbspro.2013.09.014

66. Henseler I, Regenbrecht F, Obrig H. Lesion correlates of patholinguistic profiles in chronic aphasia: comparisons of syndrome-, modality- and symptom-level assessment. *Brain*. 2014;137(3):918-930. doi:10.1093/brain/awt374

67. Ivanova MV, Zhong A, Turken A, Baldo JV, Dronkers NF. Functional Contributions of the Arcuate Fasciculus to Language Processing. *Front Hum Neurosci*. 2021;15. Accessed January 19, 2022. https://www.frontiersin.org/article/10.3389/fnhum.2021.672665

68. Geva S, Correia MM, Warburton EA. Contributions of bilateral white matter to chronic aphasia symptoms as assessed by diffusion tensor MRI. *Brain Lang*. 2015;150:117-128. doi:10.1016/j.bandl.2015.09.001

69. Rolheiser T, Stamatakis EA, Tyler LK. Dynamic Processing in the Human Language System: Synergy between the Arcuate Fascicle and Extreme Capsule. *J Neurosci*. 2011;31(47):16949-16957. doi:10.1523/JNEUROSCI.2725-11.2011

70. Forkel SJ, Thiebaut de Schotten M, Dell'Acqua F, et al. Anatomical predictors of aphasia recovery: a tractography study of bilateral perisylvian language networks. *Brain*. 2014;137(7):2027-2039. doi:10.1093/brain/awu113

For Review Only

71. Hope TMH, Seghier ML, Leff AP, Price CJ. Predicting outcome and recovery after stroke with lesions extracted from MRI images. *NeuroImage Clin*. 2013;2:424-433. doi:10.1016/j.nicl.2013.03.005

72. Laska AC, Hellblom A, Murray V, Kahan T, Von Arbin M. Aphasia in acute stroke and relation to outcome. *J Intern Med*. 2001;249(5):413-422. doi:10.1046/j.1365-2796.2001.00812.x

73. Nys GMS, Zandvoort MJEV, Kort PLMD, et al. Domain-specific cognitive recovery after first-ever stroke: A follow-up study of 111 cases. *J Int Neuropsychol Soc*. 2005;11(7):795-806. doi:10.1017/S1355617705050952

74. van der Vliet R, Selles RW, Andrinopoulou ER, et al. Predicting Upper Limb Motor Impairment Recovery after Stroke: A Mixture Model. *Ann Neurol*. 2020;87(3):383-393. doi:10.1002/ana.25679

75. Schlaug G, Marchina S, Norton A. Evidence for Plasticity in White-Matter Tracts of Patients with Chronic Broca's Aphasia Undergoing Intense Intonation-based Speech Therapy. *Ann N Y Acad Sci*. 2009;1169(1):385-394. doi:10.1111/j.1749-6632.2009.04587.x

For Review Only

Acknowledgements

The authors wish to thank Jolie Anderson, Miranda Brenneman, Cris Hamilton, Danielle Rossi, and Chia-Ming Lei for data collection. We wish to thank Erica Johns, Bowie Lin, Hao Yan, Rachel Zahn, and Riya Mehta for transcription and analysis of narrative speech samples. We thank the clinical neurological intensive care unit teams at the University of Texas Health Sciences Center and Memorial Hermann Hospital, The Houston Methodist Hospital, and the Baylor St. Luke's Hospital for their assistance in patient recruitment and neurological assessment. We gratefully acknowledge and thank our research subjects and their caregivers for their willingness to participate in this research. This work was presented at the Society for the Neurobiology of Language (2021).

Funding

This work was supported by the National Institute on Deafness and Other Communication Disorders of the National Institutes of Health under award number R01DC014976 to the Baylor College of Medicine (awarded to Schnur).

Competing interests

The authors report no competing interests.

For Review Only

Supplementary Table 1. Correlations between demographic, behavioral, lesion volume, and days tested after stroke variables and recovery in four aspects of connected speech from acute to three time points in the year following stroke.

		Acute performance	Age	Education	Lesion Volume	Days after stroke onset
subacute (n = 32)	Fluency	-0.36 (0.046)#	-0.16 (0.40)	0.01 (0.95)	0.32 (0.08)	0.24 (0.19)
	Syntax accuracy	-0.87 (1E-10)*	0.24 (0.18)	-0.12 (0.52)	0.21 (0.25)	0.35 (0.052)
	Lexical selection	-0.70 (0.000008)*	0.13 (0.50)	-0.19 (0.31)	0.21 (0.25)	-0.09 (0.61)
	Structural complexity	-0.23 (0.20)	-0.15 (0.43)	0.02 (0.94)	-0.26 (0.16)	-0.05 (0.79)
6mo (n = 31)	Fluency	-0.43 (0.02)#	0.08 (0.68)	0.31 (0.11)	0.18 (0.34)	0.27 (0.14)
	Syntax accuracy	-0.82 (2E-8)*	0.10 (0.59)	-0.25 (0.19)	0.23 (0.22)	0.29 (0.12)
	Lexical selection	-0.50 (0.004)*	0.074 (0.69)	0.004 (0.99)	0.34 (0.06)	0.03 (0.86)

For Review Only

	Structural complexity	-0.39 (0.03)[#]	0.16 (0.40)	0.17 (0.37)	-0.23 (0.21)	0.01 (0.95)
12mo (n = 25)	Fluency	-0.62 (0.001)*	0.09 (0.66)	0.07 (0.75)	0.11 (0.61)	0.02 (0.92)
	Syntax accuracy	-0.76 (0.00001)*	0.30 (0.14)	0.06 (0.79)	0.07 (0.73)	0.14 (0.52)
	Lexical selection	-0.38 (0.06)	-0.34 (0.10)	-0.13 (0.55)	0.31 (0.14)	0.41 (0.04)[#]
	Structural complexity	-0.57 (0.003)*	0.07 (0.73)	0.10 (0.66)	0.11 (0.59)	0.19 (0.36)

Numbers outside/inside parentheses denote the *r*/*p* values. *: Bonferroni correct $p < 0.05/5$; #: $p < 0.05$.

For Review Only

Supplementary Table 2. LSM results of white matter tract damage associated with decreased recovery in four aspects of connected speech from acute to three time points in the year following stroke.

		ASAF	LSAF	PSAF	FAT	IFOF	ILF	UF
subacute	Fluency	0.71	0.67	-0.55	2.79	-0.46	-0.73	-0.59
(n = 32)		(0.24)	(0.26)	(0.70)	(0.003)*	(0.66)	(0.77)	(0.72)
	Syntax	0.18	1.03	0.42	1.90	-0.64	-0.19	-1.26
	accuracy	(0.43)	(0.15)	(0.33)	(0.03)#	(0.74)	(0.58)	(0.90)
	Lexical	-0.45	0.68	0.76	-1.86	-0.52	0.11	1.48
	selection	(0.68)	(0.25)	(0.23)	(0.97)	(0.70)	(0.46)	(0.07)
	Structural	0.21	1.12	0.63	-0.61	-1.29	-1.4	-0.48
	complexity	(0.41)	(0.13)	(0.26)	(0.73)	(0.90)	(0.92)	(0.69)
6mo (n =	Fluency	-0.73	-0.41	-1.45	0.48	-0.35	-0.89	-0.54
31)		(0.78)	(0.66)	(0.92)	(0.31)	(0.64)	(0.82)	(0.70)
	Syntax	0.62	0.32	2.29	-0.94	-0.51	0.87	-1.07
	accuracy	(0.27)	(0.37)	(0.01)#	(0.83)	(0.69)	(0.18)	(0.85)
	Lexical	0.40	0.75	0.93	0.03	0.75	1.20	-0.60
	selection	(0.35)	(0.23)	(0.18)	(0.49)	(0.23)	(0.12)	(0.72)
	Structural	-0.85	0.08	-1.67	2.53	-0.19	-0.35	-0.65
	complexity	(0.80)	(0.47)	(0.95)	(0.006)*	(0.58)	(0.64)	(0.75)
12mo (n =	Fluency	1.04	0.94	0.69	-0.41	-1.36	-0.67	-1.09
25)		(0.15)	(0.18)	(0.24)	(0.66)	(0.92)	(0.92)	(0.86)

For Review Only

Syntax	1.04	2.28	0.75	-0.36	-1.38	0.32	-2.07
accuracy	(0.15)	(0.01)#	(0.23)	(0.65)	(0.92)	(0.37)	(0.98)
Lexical	-0.38	-0.42	-0.03	0.47	0.49	-0.37	0.79
selection	(0.65)	(0.66)	(0.51)	(0.32)	(0.31)	(0.65)	(0.21)
Structural	-1.21	-0.50	-2.24	2.57	-0.23	-1.06	0.85
complexity	(0.89)	(0.69)	(0.99)	(0.004)*	(0.59)	(0.86)	(0.20)

Numbers outside/inside parentheses denote the *z/p* values. *: Bonferroni correct $p < 0.05/7$; #: $p < 0.05$. ASAF: anterior segment of arcuate fasciculus; LSAF: long segment of arcuate fasciculus; PSAF: posterior segment of arcuate fasciculus; FAT: frontal aslant tract; IFOF: inferior fronto-occipital fasciculus; ILF: inferior longitudinal fasciculus; UF: uncinate fasciculus.

Supplementary table 3. The LSM p values controlling for degree of gray damage at tract origins and termini gray matter ends' damage.

	subacute: FAT-syntax	subacute: FAT-fluency	6mo: FAT-sentence structure	6mo: PSAF-syntax	12mo: FAT-sentence structure	12mo: LSAF-syntax
P values	**0.028**	**0.011**	**0.018**	0.081	**0.012**	**0.019**

Bold font indicates p < 0.05.

For Review Only

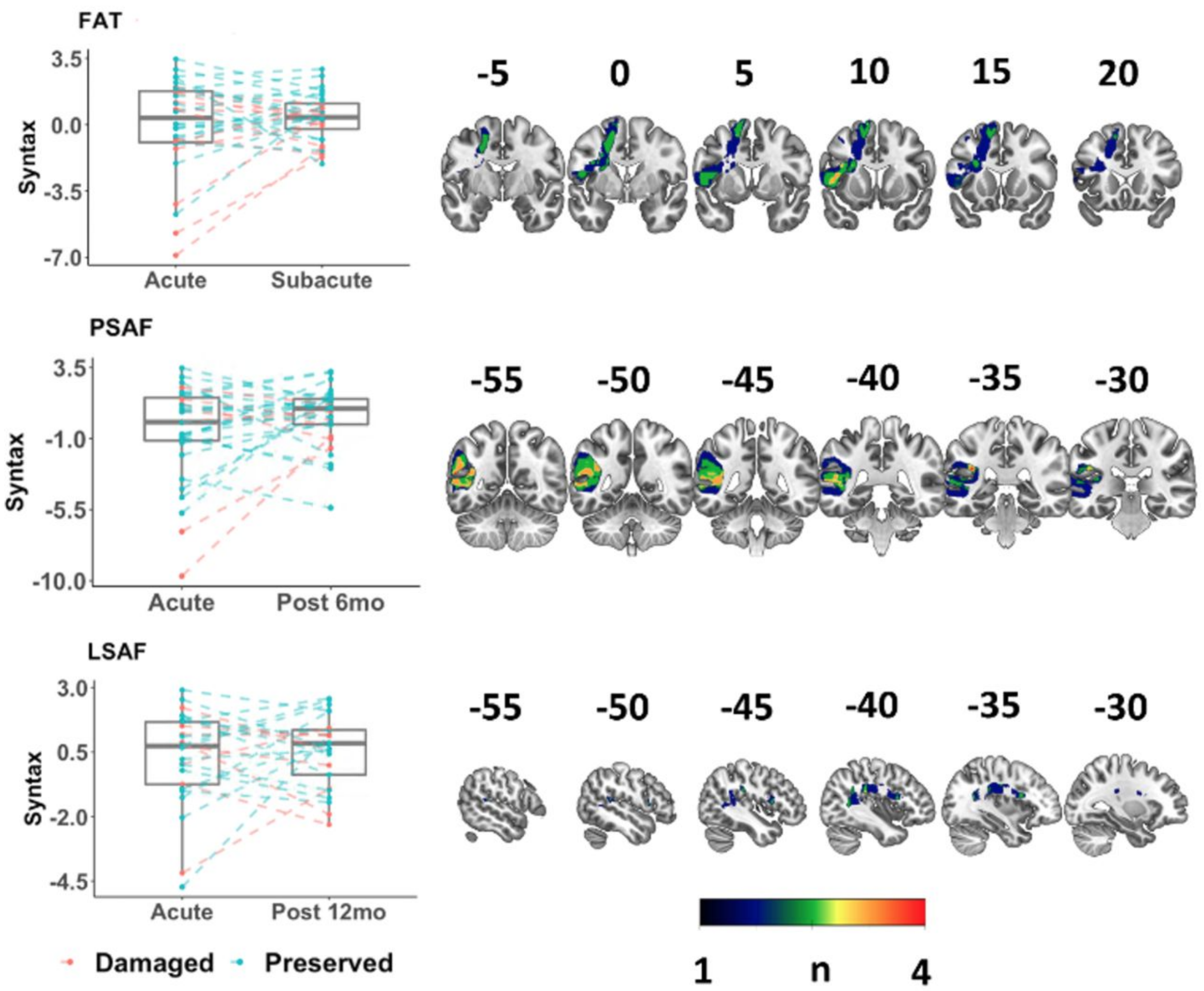

Supplementary Figure 1. The white matter tracts whose acute damage significantly predicted decreased recovery in four aspects of connected speech across three time points in the year following stroke (subacute, 6mo, and 12mo post stroke) without correction for multiple comparisons. The box plots show the acute and follow-up connected speech scores clustered by tract damage. The brain maps show lesion overlaps of the damaged group on the corresponding tracts. Red lines indicate subjects with damaged tracts, while green lines indicate subjects with spared tracts. The numbers above the brain coronal and sagittal slices indicate their MNI y and x coordinates, respectively.
