# Supplementary material for: Anterior connectivity critical for recovery of connected speech after stroke
Source: Brain Commun. 2022 Oct 19;4(6):fcac266. doi: 10.1093/braincomms/fcac266 (PMC9651028; doi:10.1093/braincomms/fcac266)
Supplement: fcac266_Supplementary_Data [file Original Submission - Author's Response.pdf › Original Submission - Author's Response.pdf]

We appreciate the reviewers' careful reading of the manuscript and the constructive critiques. Below are the reviewers' comments (in italics) and our responses. In the manuscript, sections that were changed are in colored text, and referred to below by page number.

Reviewer 1

1. *The authors report an impressive study of 41 patients studied at the acute stage of recovery and/or at later stages (2, 6, and 12 months post-stroke) on novel measures of connected speech and MRI to identify damage to specific white matter tracts that affect recovery of various aspects of connected speech. The paper is well-written and most of the methods are sound.*

We were pleased to hear that the reviewer felt that the paper was easy to read and well-written!

2. *The major weakness of the study is one that they identify themselves: "To maximize power to detect factors which affected recovery, we included the largest number of subjects tested both acutely and at a single subsequent time point. Although this approach helped avoid type II statistical errors, i.e. false negatives, it rendered direct comparisons between time points difficult to interpret."*
   In fact, it makes all of the conclusions about the effect of lesions at one point on recovery at a later point impossible to interpret, because the patients are different patients. This would be a fatal flaw unless the authors re-analyzed their results using only the subset of patients who were tested at the same time points. Ideally, they would want to include only patients tested at ALL time points, especially for comparisons across the time points, like figures 3 & 4 and Table 1. These figures and tables do not make any sense, since each time point represented different subsets of patients. However, clearly that is not possible to do the ideal approach (and understandably so, as patients simply are unable to be tested at certain time points for various reasons). At the very least, they would need to make separate tables and figures that include the same subset of patients at each time point.

DONE. We now include both the original longitudinal analyses (acute and either 1mo, 6mo, or 12mo timepoints), and the requested reanalysis of our results using only the subset of patients who were tested at ALL four time points (n=20; see pp. 9, 11, 14). We report new Tables (new versions of Table 1 and SI Table 4 with n=20 across all time points, see SI Tables 1 and 5; pp. 35 & 41) and new figures (new versions of Figures 2, 3 & 4 with n=20 across all four time points, see SI Figures 2, 3 and 4; pp. 44-46). When we examined performance in the 20 subjects who were able to return across all four time points, the pattern of results supported the larger analysis regarding associations between acute white matter damage and recovery of connected speech performance and was significant at the $p =< .05$ threshold. Specifically, acute frontal aslant tract damage (FAT) damage continued to be associated with decreased structural complexity at the chronic stages of stroke (6 and 12 months after stroke). Of note however, at the subacute stage, acute FAT damage was no longer associated with decreased recovery of fluency. The change in association between fluency and acute FAT damage may have occurred in part because of the multidimensionality of fluency. Because fluency is dependent on multiple capacities (e.g., articulation, phonology, comprehension, working memory; cf. Nozari & Faroqi-Shah, 2017), after stroke, a speaker will have reduced fluency because of a deficit in any or all of these capacities. By removing the participants unable to return across time points, we may have removed a significant proportion of those with a common underlying cause of disfluency associated with acute FAT damage. We discuss these new analyses in the methods (pp. 9, 11, 14), results (pp. 15, 18, 20), and general discussion (pp. 23-25, 27).

New SI Figures 3, 4 and SI Tables 1 and 5 included below (see response to Reviewer 2 point 3 for SI Figure 2).

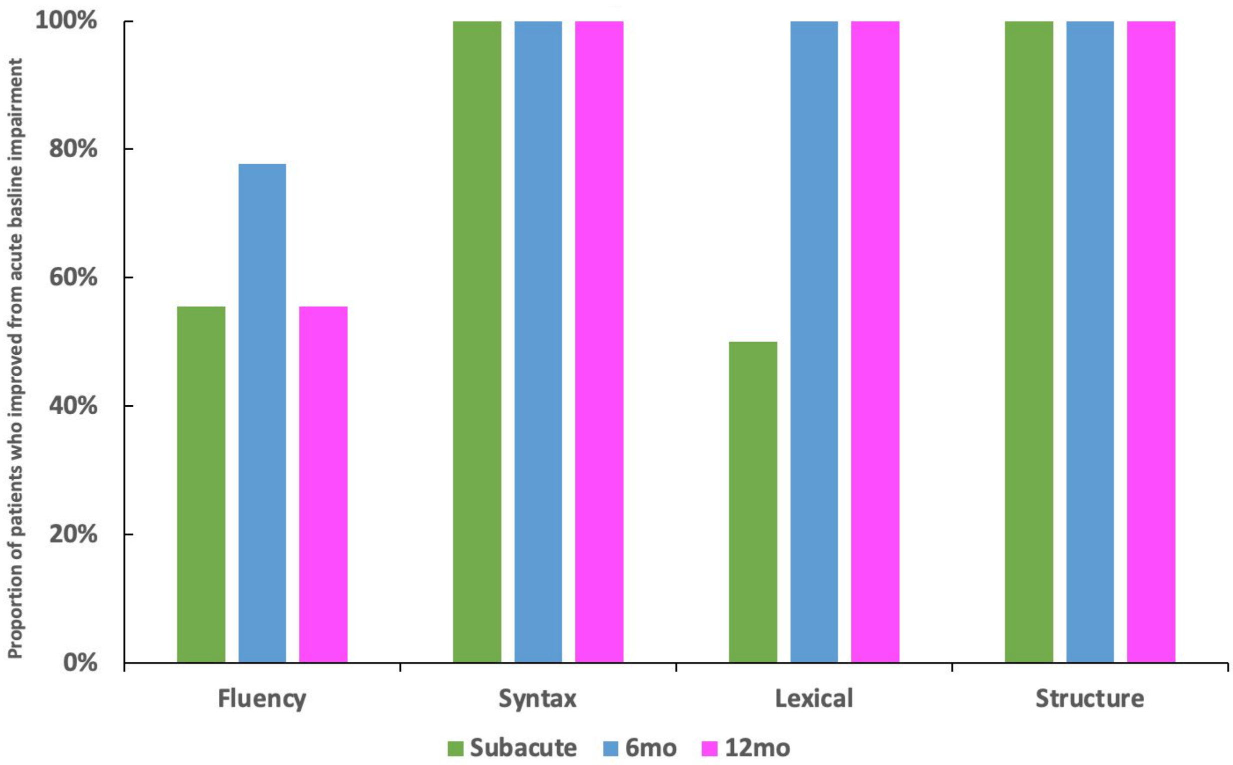

SI Figure 3. The proportions of patients tested at all three time points in the year following stroke (subacute, 6 months, 12 months; n = 20) who recovered to within normal levels (*z* > -1.67) after beginning with impairment acutely (*z* < -1.67) in four aspects of connected speech.

SI Table 1. The numbers and proportions of patients tested at all three time points in the year following stroke (subacute, 6 months, 12 months; n = 20) who recovered to within normal levels (*z* > -1.67) after beginning with impairment acutely (*z* < -1.67) in four aspects of connected speech.

	Fluency	Syntactic accuracy	Lexical selection	Structural complexity
Subacute (n = 20)	5/9 (56%)	3/3 (100%)	1/2 (50%)	1/1 (100%)

6mo (n = 20)	7/9 (78%)	3/3 (100%)	2/2 (100%)	1/1 (100%)
12mo (n = 20)	5/9 (56%)	3/3 (100%)	2/2 (100%)	1/1 (100%)

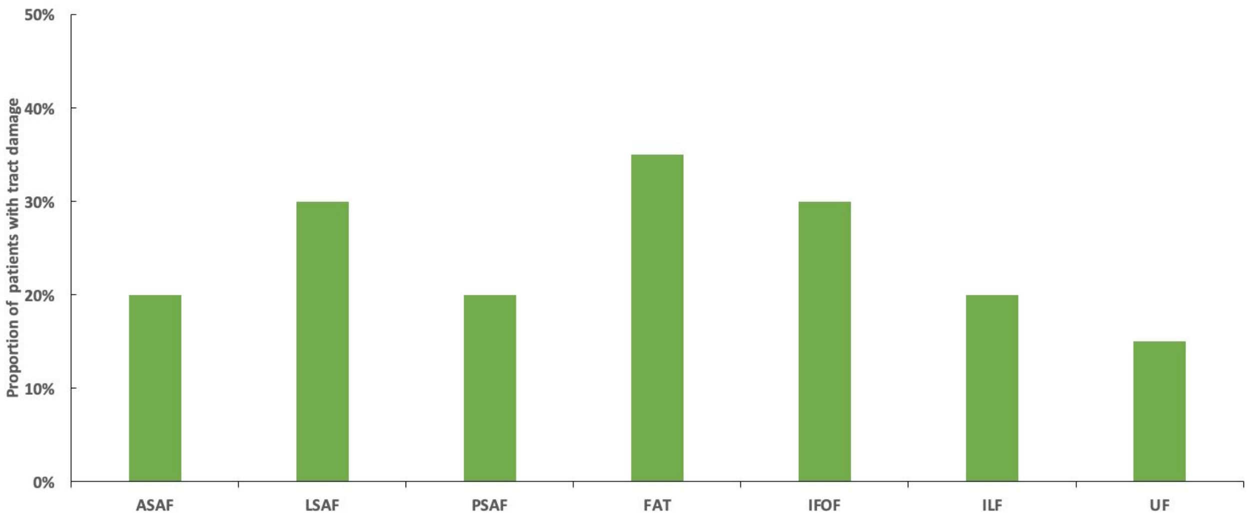

SI Figure 4. Proportions of patients with acute tract damage for each tract in the patient cohort who were tested at all three time points in the year following stroke (subacute, 6 months, 12 months; n=20).

SI Table 5. LSM results of acute white matter tract damage associated with decreased recovery in four aspects of connected speech for patients tested acutely, and all the three time points in the year following stroke (subacute, 6 months, 12 months; n = 20).

		ASAF	LSAF	PSAF	FAT	IFOF	ILF	UF
subacute	Fluency	-0.57	-0.57	-0.40	0.88	-1.33	-0.88	-0.66
(n = 20)		(0.71)	(0.72)	(0.66)	(0.19)	(0.90)	(0.81)	(0.75)
	Syntactic	-0.54	0.66	0.32	0.79	-1.57	-0.36	-1.86
	accuracy	(0.71)	(0.25)	(0.37)	(0.21)	(0.93)	(0.65)	(0.92)

	Lexical selection	0.93 (0.18)	1.59 (0.07)	1.71 (0.07)	-0.67 (0.76)	1.47 (0.08)	2.23 (0.05)#	3.03 (0.04)#
	Structural complexity	0.30 (0.38)	0.95 (0.17)	0.46 (0.32)	0.40 (0.35)	-0.91 (0.82)	-1.40 (0.89)	-2.66 (0.95)
6mo (n = 20)	Fluency	-0.93 (0.82)	-0.37 (0.65)	-0.31 (0.62)	-0.78 (0.78)	-1.21 (0.88)	-2.16 (0.95)	-0.28 (0.62)
	Syntactic accuracy	0.68 (0.24)	0.43 (0.34)	2.05 (0.05)	-0.24 (0.59)	0.08 (0.46)	0.37 (0.35)	-0.31 (0.62)
	Lexical selection	1.55 (0.09)	1.46 (0.09)	1.20 (0.13)	-0.61 (0.73)	4.42 (0.003)*	2.25 (0.04)#	2.42 (0.06)
	Structural complexity	-0.15 (0.56)	0.21 (0.41)	-1.40 (0.89)	2.46 (0.02)#	0.33 (0.37)	0.44 (0.32)	-0.97 (0.81)
12mo (n = 20)	Fluency	0.85 (0.20)	0.77 (0.22)	0.57 (0.28)	-0.11 (0.55)	-1.40 (0.91)	-1.13 (0.86)	-0.68 (0.75)
	Syntactic accuracy	1.16 (0.14)	2.54 (0.02)#	1.02 (0.16)	0.04 (0.49)	-0.71 (0.76)	0.48 (0.31)	-1.68 (0.90)
	Lexical selection	0.17 (0.43)	0.16 (0.44)	0.55 (0.29)	-0.28 (0.62)	0.27 (0.39)	0.20 (0.42)	-0.23 (0.60)
	Structural complexity	-1.04 (0.84)	-0.52 (0.70)	-2.94 (0.98)	2.14 (0.03)#	-0.32 (0.63)	-0.76 (0.78)	0.34 (0.37)

Numbers outside/inside parentheses denote the *z/p* values. *: Bonferroni correct $p < 0.05/7$; #: $p < 0.05$. ASAF: anterior segment of arcuate fasciculus; LSAF: long segment of arcuate fasciculus; PSAF: posterior segment of arcuate fasciculus; FAT: frontal aslant tract; IFOF: inferior fronto-occipital fasciculus; ILF: inferior longitudinal fasciculus; UF: uncinate fasciculus.

Other suggestions

1. *Please report test-retest and inter-rater reliability for the connected speech analyses.*

   DONE. The participants' connected speech quantitative production analysis (QPA) variables we used in this study were calculated by two raters whose inter-rater reliabilities for transcription, utterance segmentation, and QPA variable extraction were originally reported in Martin and Schnur (2019). Transcription and scoring inter-rater reliabilities were high (average 95% and 93% agreement, respectively). We now provide details on p. 10. To provide an estimate of test-retest reliability for the QPA derived factor scores, we correlated performance across the 6- and 12-mo

timepoints in a subset of 14 participants who performed within control levels of connected speech (> -1.67 SDs of controls). Performance significantly correlated across timepoints for factor scores representing structural complexity, lexical selection, and fluency (r's > 0.59; p's < 0.03). Syntactic accuracy did not reach significance likely due to variability in individual syntactic accuracy recovery during later stages of stroke (r=-0.27, p = 0.36). See details on p.11 .

2. *Although it is tempting to describe "statistically significant" results that do not survive correction for multiple comparisons, these results should at most be reported in the supplemental materials, as they could be due to chance alone.*

   DONE. See supplemental materials p. 35.

3. *You report, "Three patients were recruited with neurological signs of acute LH stroke but no clear lesion was identified from neuroimaging." Why were these patients included? They were likely patients with stroke mimics, so they would not be appropriate for studying disruption of white matter tracts that affect connected speech (or its recovery). These patients should be removed, and the data re-analyzed.*

   The three patients we included with neurological signs of acute stroke whose lesions were difficult to identify on neuroimaging were not stroke mimics. These participants were diagnosed at hospital admission with acute cerebral vascular accidents (CVA) and two were administered the clinical intervention tissue plasminogen activator (tPA) which reduced the impact of the CVA on imaging. However, one patient was not administered tPA, did not have an observable lesion, and had a record of TIAs. To comply with the reviewer's strict criterion, we excluded this person and reanalyzed the data. There were no changes in the results. See pp. 9, 14, 20 and Table 1 for methods and results with the revised subject sample.

4. *Despite the limitations, there are some interesting findings such as, "During the subacute stage after stroke, acute FAT damage led to decreased recovery of fluency (z = 2.79, p = 0.003) and at the chronic stages (six – 12 months post- stroke) significantly decreased recovery of structural complexity (z values > 2.53, p values < 0.006) (see Figure 4)." However, the sentence would be much easier to read as separate sentences, especially since the 2 parts of the sentence are referring to different subsets of patients. For example, you could say, "Acute FAT damage led to decreased recovery of fluency at the subacute stage after stroke, (z = 2.79, p = 0.003). In an overlapping subset of patients, acute FAT damage led to significantly decreased recovery of structural complexity at the chronic stages (six – 12 months post- stroke) (z values > 2.53, p values < 0.006)."*

DONE.

5. *It is surprising that lesion volume did not predict the degree of connected speech recovery at any time point in the year after stroke even though initial severity did, since lesion volume and severity are generally highly correlated. Is this still true if you remove the 3 patients who had no visible lesions?*

Lesion volume did not predict degree of connected speech recovery at any time point in the year after stroke whether we removed one subject with a history of TIAs (p's > 0.06, see our response to *Other suggestions* Point 3), or removed all three subjects with no observable lesions (p's > 0.07). In addition, we calculated correlations between lesion volume and initial severity. Acute fluency significantly related with lesion volume (SI Table 3: r=-0.36, p=0.03; other factors: p's > 0.10). We discuss these results as indicating that lesion volume predicts deficit severity, but not recovery (see pp. 19, 26).

Minor Suggestions

1. *p. 3 "…facilitate language intervention, it is important to identify factors which predict degree of connected speech recovery soon after stroke" should be changed to, "… facilitate language intervention, it is important to identify factors THAT predict degree of connected speech recovery soon after stroke."*

   DONE.

2. *p.3 "Damage to disparate brain regions and their connections impairs word retrieval and its recovery but the neural predictors of the recovery of connected speech are unknown" requires a comma after recovery.*

   DONE.

3. *p.5 "It is unclear why damage to white matter tracts which connect different parts of the language network associated with different language functions have not been strongly differentiated with regards to connected speech," should be changed to, "It is unclear why damage to white matter tracts THAT connect different parts of the language network associated with different language functions have not been strongly differentiated with regards to connected speech."*

   DONE.

Reviewer 2

1. *My main concern is sample size. Although it initially sounds like you have a good number of participants, particularly for a longitudinal study, in Figure 5 where you highlight individuals in red who have damage to the FAT, this looks like a very small proportion of the participant total. This is highlighted again in your brain maps, where even the largest overlap in participants is just 4 people. This paper would benefit from more participants with damage to the FAT, in order to support your conclusions.*

DONE. Through a misunderstanding due to the poor presentation of Figure 5, Reviewer 2 suggested our study included too small a sample size of participants with damage to the frontal aslant tract (FAT) at 4 out of 32 participants (13% proportion). In reality, the lowest proportion of participants with damage to the FAT across time points was 9 of 25 patients, a 36% proportion, and the highest proportion of patients with damage to the FAT was 42%. For the n=20 subsample, 7 out of 20 patients had damage to the FAT, a 35% proportion (see response to R1's point 2). Thus, with a close to even split of participants

with damage to the FAT (36%-42%), our conclusions are well-supported. Figure 5's brain map of damage to different white matter tracts misled the reader. This is because Figure 5 depicted where patients had lesions *within* the FAT and lesion damage within the FAT did not 100% overlap in voxels across patients. Thus, although a minimum of 9 people had damage to the FAT, identical voxels damaged across the entire FAT extent occurred in 4 participants. To avoid further misleading, we removed the brain map from Figure 5. We clarified this point both in the text (see p. 18) and included an updated Figure 5 (p. 21).

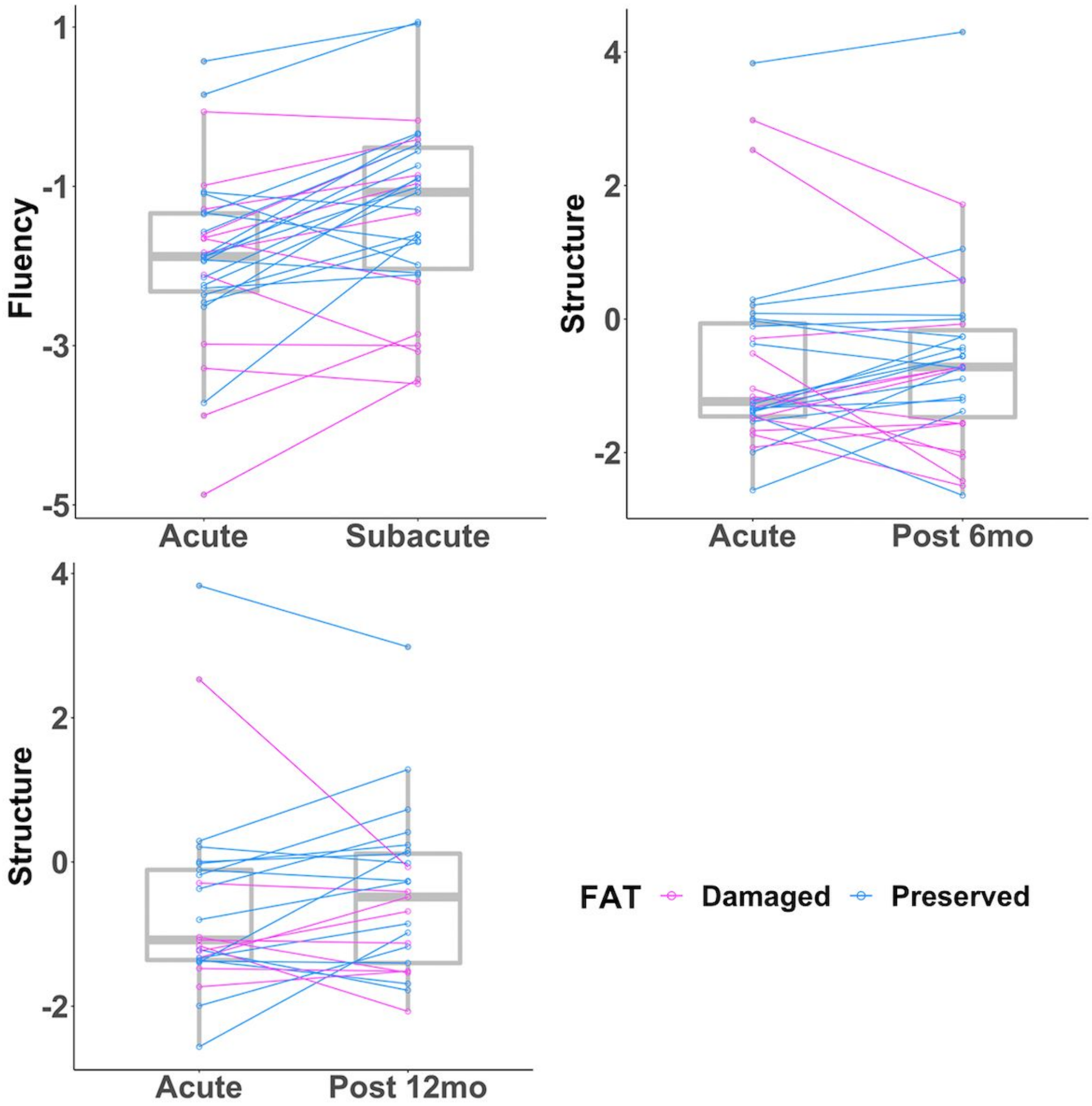

Figure 5. The white matter tracts whose acute damage predicted decreased recovery in four aspects of connected speech from the acute stage to three time points in the year following stroke (subacute, 6mo, and 12mo post stroke) after multiple-comparison correction (p < 0.05/7).

2. *In the Introduction, although you do cite relevant literature, more detail could be added about papers which have found similar results - such as Alyahya et al., 2020 who also uses a lesion symptom mapping approach with stroke patients and highlighted the role of the FAT in the chronic stage. Although this manuscript builds on this with the addition of multiple timepoints, including the acute stage, better description of studies such as this in the Introduction would help the reader to understand your motivation for focusing on tracts such as the FAT.*

DONE. To address the concern that we do not clearly describe why we focused on specific white matter tracts like the FAT, we amended the "Current Study" section to more clearly explain our rationale, p. 8: "We intentionally focused on white matter tracts which connect gray matter regions previously found to be involved in language production and also additional segments of the AF (i.e. ASAF and PSAF)." We also corrected our omission in the introduction of the Alyahya et al. (2020) findings, on p. 5: "We are aware of one study where lesion damage associated with the reduced number, diversity, and speed of content words produced during picture descriptions and procedural discourse, overlapped with atlas-defined FAT (along with the LSAF and the ASAF) in a group of 46 speakers with chronic aphasia[3]. These results point to a potential role for the FAT in lexical retrieval and fluency, but statistical evidence of this tract's function is needed."

3. *On Figure 2, I understand that you are using performance within the normal range as a way to measure recovery, which is interesting. However, in this figure there are clearly individuals who are colored red (i.e., worse recovery) who do make large gains, albeit not to control range, and it concerns me that you are not addressing this.*

DONE. We are pleased to see Reviewer 2's appreciation of how recovery to within unimpaired performance is interesting, especially from a patient's perspective as it is relevant to quality of life. We thank Reviewer 2 for in addition, pointing out that a substantial increase in recovery, even if not to within unimpaired performance levels, is also important to acknowledge. To address this point, we extended the discussion of connected speech recovery to also include participants who experienced significant recovery (recovery > 1 SD) but not to within unimpaired levels, see p. 15: "When considering those participants who made large gains (recovery > 1 SD) but not to within the control range (see SI Figure 1), the recovery patterns were relatively consistent with the group of participants who recovered to within control levels. Specifically, syntactic accuracy, structural complexity and lexical selection patterns of recovery were similar. However, for fluency when including participants who made large gains but outside the control range, 70% of impaired participants improved during the year after stroke (in comparison to 50% of participants who improved to within the control range)." We now include an updated SI Figure 1 (shown below), adding this new recovery trajectory in yellow.

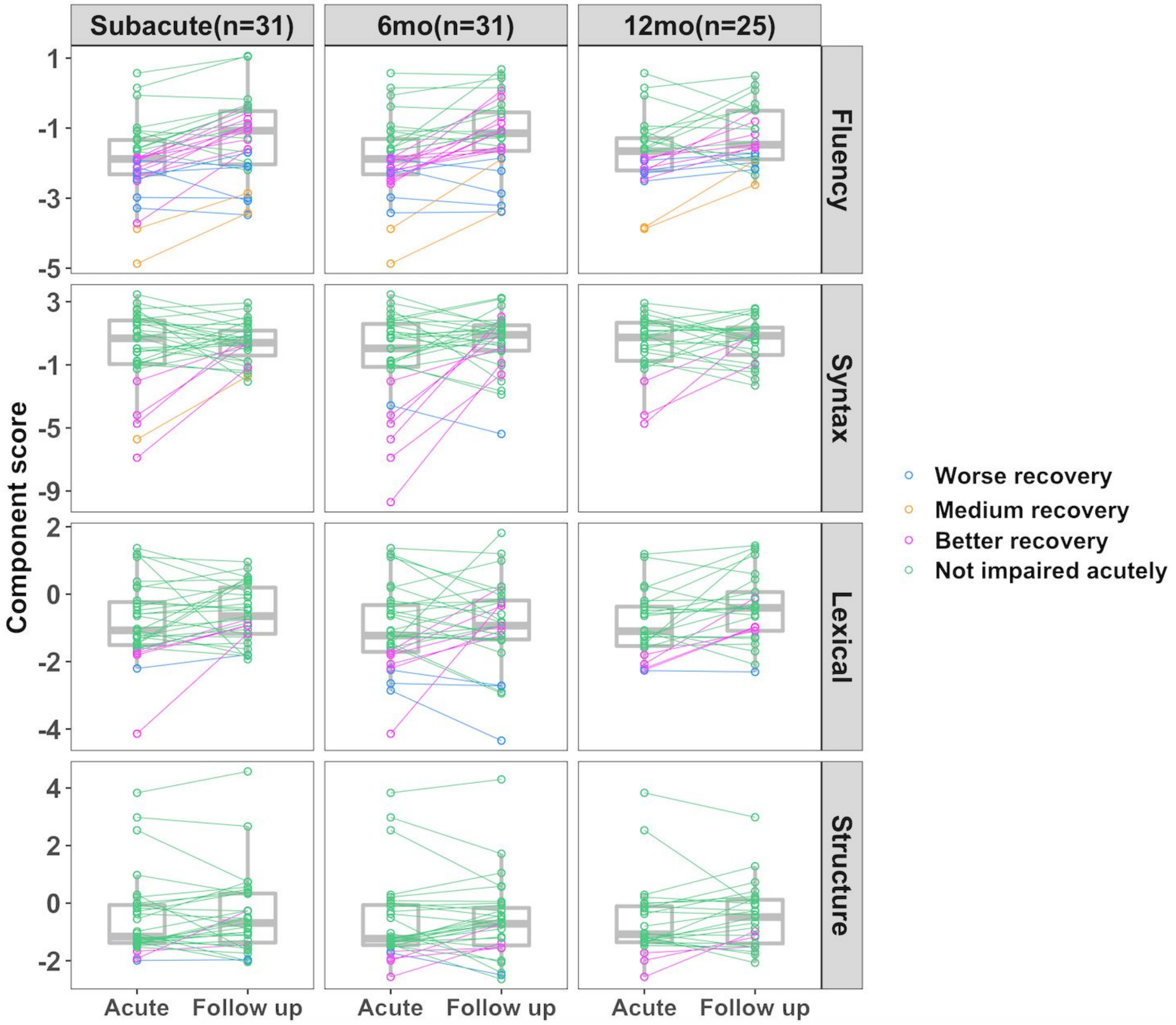

SI Figure 1. Different recovery trajectories of four aspects of connected speech from acute to three follow-up time points in the year following stroke. Worse recovery was defined as both acute performance worse than 95% of controls' performance (z's < -1.67) and follow up performance still worse than 95% of controls (z's < -1.67; in blue). Medium recovery was defined as acute performance worse than 95% of controls' performance (z's < -1.67) where large gains occurred (recovery > 1 SD) but not to within the control range (in yellow). In contrast, better recovery was defined as those with impaired acute performance z's < -1.67 but subsequent follow up performance was better than 95% of control performance (z's > -1.67; in magenta). We defined not impaired as individuals who performed better than 95% of controls' performance acutely (z's > -1.67; in green).

3. [continued…] It would also be good to see comparisons between subacute-6mo, and 6mo-12mo, because it is difficult to see whether all the gains are in the acute- subacute stage, or if there are some individuals who have gains later into their recovery. The figure is also a little small, making it difficult to see individual trajectory.

To address the difficulty of seeing whether all the gains are in the acute-subacute stage, or whether there are some individuals who have gains later into their recovery, we now include a supplemental figure of recovery for individuals tested across all timepoints (n=20; SI Figure 2, p. 44; also see response to R1, point 2). Here, individual patterns of recovery can be seen from acute to 1 month, 1-6 and 6-12 months. To better see individual recovery trajectories, we also increased the size of both the original Figure 2 and the new SI Figure 2.

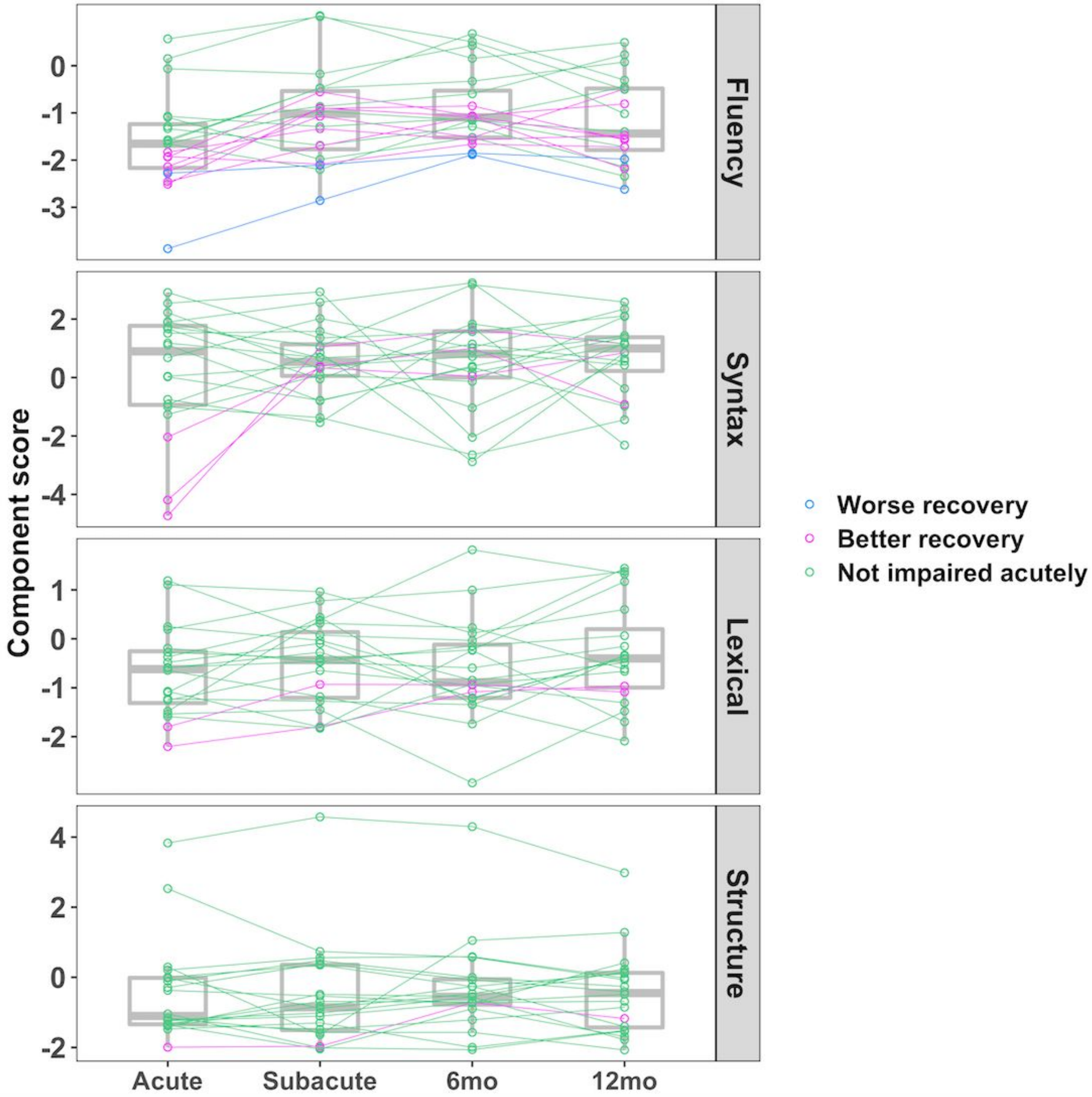

SI Figure 2. Recovery of four aspects of connected speech for the 20 patients testing from acute to three follow-up time points in the year following stroke

4. *On Figure 4, it is good to see the proportion of individuals with damage for each tract, but this would be much more informative if we could see the proportion of damage for each individual. If one participant only has damage to 10% of the tract, we might expect very different implications than for someone with 100% tract damage.*

To address R2's interest in seeing the proportion of damage for each individual for each tract, we added SI Table 2 (p. 36) to show the summary of individual's tract damage proportion.

SI Table 2. Summary of average acute damage proportion for each tract across patient cohorts at subacute, early chronic (6mo), and late chronic (12mo)time points.

Tract	Subacute (n = 31)	6mo (n = 31)	12mo (n = 25)	All time points (n=20)
ASAF	2%±4% (0-16%)	2%±4% (0-16%)	1%±2% (0-8%)	1%±2% (0-8%)
LSAF	2%±5% (0-23%)	3%±6% (0-23%)	1%±3% (0-10%)	1%±3% (0-10%)
PSAF	2%±9% (0-50%)	4%±12% (0-50%)	2%±6% (0-30%)	1%±1% (0-5%)
FAT	2%±5% (0-24%)	2%±5% (0-24%)	2%±5% (0-24%)	2%±6% (0-24%)
IFOF	1%±3% (0-11%)	1%±2% (0-11%)	1%±2% (0-11%)	1%±3% (0-11%)
ILF	1%±4% (0-16%)	1%±4% (0-16%)	1%±3% (0-16%)	1%±4% (0-16%)
UF	1%±2% (0-14%)	1%±2% (0-14%)	1%±3% (0-14%)	1%±3% (0-14%)

Numbers indicates Mean±SD (range).

4. [continued…] *You also don't provide any information in the discussion regarding why fewer individuals have damage to these tracts at the later timepoints, which would be a good addition.*

Because overlapping but different groups of participants were tested at each follow-up time point, the proportion of participants with damage to a tract changed depending on the group composition. However, although group composition varied across timepoints, the proportions of participants with damage to any particular tract did not substantially change across subgroups of participants returning at either 1mo, 6mo, and 12mo as evidenced by high overlapping proportions of subjects between time points (subacute-6mo: 84%; subacute-12mo: 75%; 6mo-12mo: 79%). Nevertheless, we acknowledge that the 12mo group has fewer people and fewer people with damaged tracts (see Figure 4, p. 18). We speculate patients with more severe tract damage proportion were more likely not to attend further follow-up studies, probably because they suffer more difficulties due to the more severe damage. We discuss this on p. 26.

5. *In Supplementary Table 1 you include correlations with different variables at each of the follow up timepoints, but it would be useful to see whether there are correlations with acute performance, particularly between acute performance and lesion volume.*

DONE. See new SI Table 3, first four rows (p. 37).

6. *Throughout the paper, it can be difficult to know which subset of patients you are referring to. For example on page 21, line 24, and page 22, line 8, you mention 80% of participants recovering, but does this include individuals who were initially in the control range? It would be good if you could be more specific throughout.*

DONE. Thank you for bringing to our attention this confusion. We amended the discussion on pp. 14-15 and 23-24 to clarify that the proportions of participants recovering by 1, 6 and 12 months post-stroke included those participants who were classified as initially having impairments at the acute stage of stroke and did not include individuals who were initially in the control range.

7. *Finally, some of your methods could use more explanation for the motivation behind them. For example, why you chose to use results from a previous PCA rather than raw results, or why you chose to z-score the behavioral results rather than directly compare raw change, given that the same participants were used throughout. More detail of your motivation would help the reader to understand more about the methods, and why this might be a better/worse approach than other (more simple) options.*

Thank you for this helpful suggestion to clarify our approach. We used the results from a previous PCA (Ding et al., 2020) because it included a larger sample size (n=65) and thus the resulting derived principle components are more reliable than using components derived from a smaller subject sample (here, n= 40). We used z scores instead of raw scores because we could more easily compare the different PCA factors. This also helped to interpret the degree of deficit more intuitively in comparison to using raw scores. We now discuss these points in the methods (see p. 10).

8. *Overall, I think this is an excellent endeavor, and the results seem promising, particularly as they seem to align with results from the chronic stroke literature. However, I think the conclusions are a little strong, given the small proportion of individuals with damage to the regions of interest.*

We very much appreciate Reviewer 2's assessment that this project was an excellent endeavor with promising results. We believe with the clarification that we did in fact include a sizeable proportion of individuals with damage to the regions of interest (see response to Reviewer 2, Point 2), we now have mitigated concerns regarding the strength of the conclusions drawn from the data.

Scientific Editor

1. *Fig4: please report in the figure legend the test used and relevant test statistic values alongside p values (for example F or t values for ANOVAs and t-tests).*

We did not perform any statistical tests in Figure 4.

2. *Fig2 and Fig5: please, replace red colour with magenta to make the figure color blind friendly.*

DONE

3. *Fig3-4: please, replace yellow colour with magenta to make the figure color blind friendly.*

DONE

4. *Please include a paragraph about statistical analysis in the 'Materials and Methods' section.*

DONE. See pp. 13-14.

5. *Please supply a graphical abstract. A graphical abstract is a visual representation of the central finding or methodology of the paper. It will be displayed in the table of contents. Authors should strive to make the graphical abstract informative, interesting, visually appealing, and straightforward (no caption needed, no abbreviations except gene names). Please upload as a separate high resolution image file (tiff preferred). The technical requirements for the graphical abstracts are:*

--size of the submitted image: 1200 pixels square at 300dpi

--font of the text: Arial 12-16 point (smaller fonts will not be legible online)

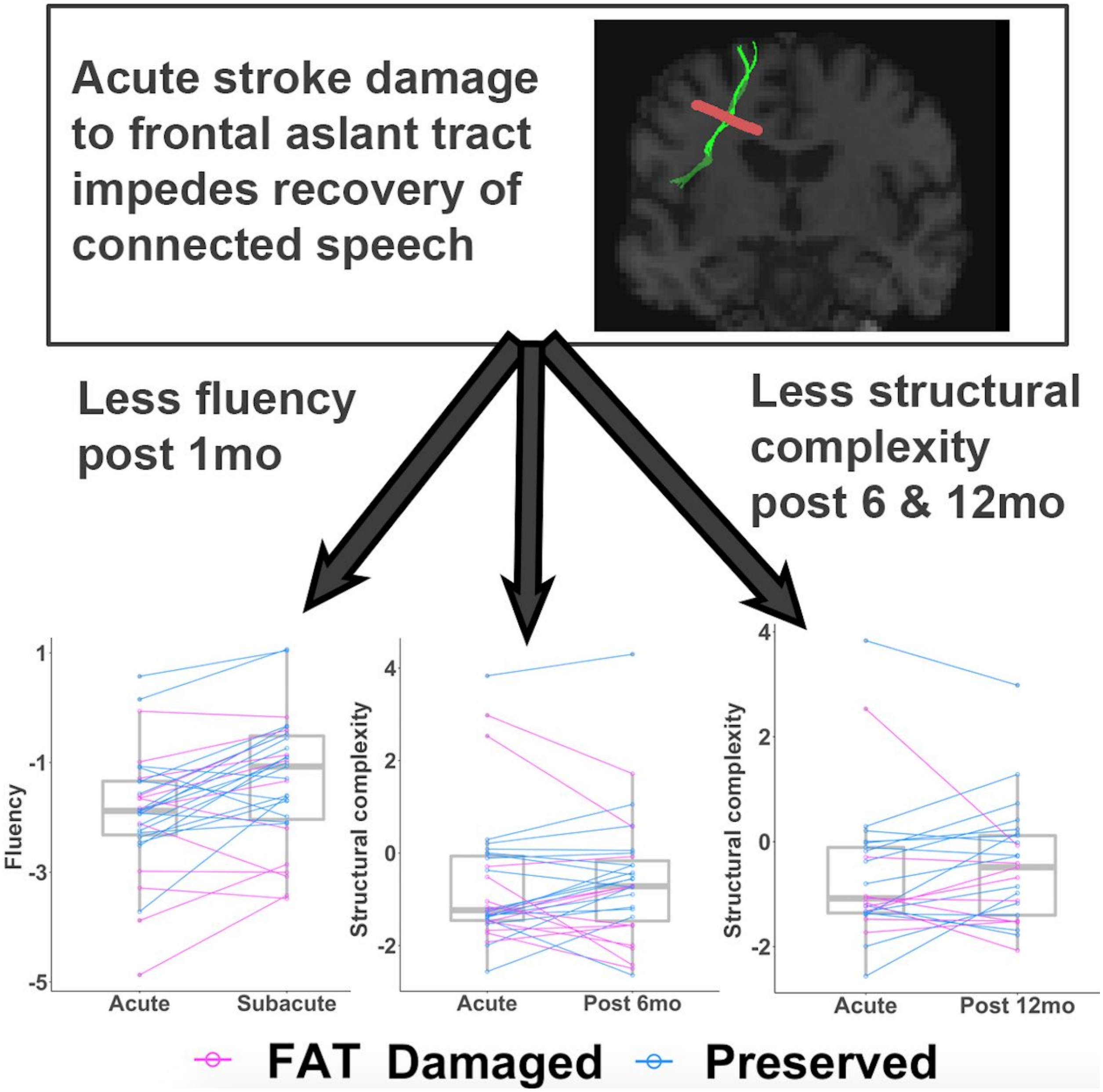

6. *Please add an Abbreviated Summary (up to 50 words) of your paper that captures the main purpose and conclusions of the work. The summary should state the main results written in the*

third person (‘[1st author surname] et al. report that…’) and conclusion. This summary will be used in the contents list online and should not contain any abbreviations other than those on the accepted abbreviations list and accepted gene/protein names, making it accessible to the non-specialist. Please upload it as a Word document. Allowed abbreviations: AIDS; ANOVA; AMPA; ATP; A,T,C,G; CNS; CSF; CT; DNA; ECG; EEG; EMG; GABA; HIV; MRI; NMDA; PET; PCR; RNA.

DONE.
