# Supplementary material for: Anterior connectivity critical for recovery of connected speech after stroke
Source: Brain Commun. 2022 Oct 19;4(6):fcac266. doi: 10.1093/braincomms/fcac266 (PMC9651028; doi:10.1093/braincomms/fcac266)
Supplement: fcac266_Supplementary_Data [file Original Submission - Decision Letter.pdf › Original Submission - Decision Letter.pdf]

Original Submission - Decision Letter

07-Mar-2022

Dear Dr. Schnur,

Thank you for your recent submission BRAINCOM-2022-035 entitled "Anterior Connectivity Critical for Recovery of Connected Speech after Stroke" to Brain Communications.

Your manuscript has been reviewed, and while the reviewers and editorial team appreciate many aspects of the paper, they raise some concerns that you will need to address in order for the paper to be considered for publication. In particular, both reviewers raised important methodological concerns, such as low sample size and the need to show in the tables and text the same subset of patients at each time point in order to facilitate data interpretation.

If you can address these concerns, I invite you to submit a revised manuscript and a response to each of the reviewers' comments. The reviewers' comments are included at the bottom of this message.

In addition to the reviewers' comments, please also address our Scientific Editor's comments added below to comply with our requirements for presentation of scientific data at Brain Communications.

Please format your revised manuscript according to our Instructions to Authors https://academic.oup.com/braincomms/pages/General_Instructions – a summary of key points is attached to help with this.

To revise your manuscript, click the link below or log into https://mc.manuscriptcentral.com/braincom and enter your Author Centre, where you will find your manuscript title listed under "Manuscripts with Decisions." Under "Actions," click on "Create a Revision." Your manuscript number will be appended to denote a revision.

*** PLEASE NOTE: This is a two-step process. After clicking on the link, you will be directed to a webpage to confirm. ***

https://mc.manuscriptcentral.com/braincom?URL_MASK=5c7627ff5948405aae1841fca56fe1c7

Please upload a revised manuscript with changes tracked with track changes or in coloured text.

When submitting your revised manuscript, you will be asked to respond to the reviewer(s) comments. Please be as specific as possible in your response to all of the comments.

The editorial team at Brain Communications aims to increase transparency in scientific publishing and to demonstrate the value that peer review brings to the scientific process. Please therefore consider whether you are willing to have reviewer(s)' comments on your paper and your response published as supplementary data with the paper if it is accepted. This is entirely optional and will only be done if both the authors and reviewers agree. While submitting your revision there will be an opportunity to review or update the answer you gave previously, if necessary.

IMPORTANT: Your original files are available to you when you upload your revised manuscript. Please delete any redundant files before completing the submission.

Because we are trying to facilitate timely publication of manuscripts submitted to Brain Communications, we would appreciate your revised manuscript as soon as possible. If it is not possible for you to resubmit within 3 months, the option to submit a revision will expire, and we would then consider your revised paper as a new submission. However, if you need a little more time, please get in touch and we can extend this deadline for you.

Once again, thank you for submitting your manuscript to Brain Communications and I look forward to receiving your revision.

Best wishes

Dr. Alberto Lleo
Associate Editor, Brain Communications

Professor Tara Spires-Jones
Founding Editor, Brain Communications

Reviewer(s)' Comments to Author:

Reviewer: 1

Comments to the Author
The authors report an impressive study of 41 patients studied at the acute stage of recovery and/or at later stages (2, 6, and 12 months post-stroke) on novel measures of connected speech and MRI to identify damage to specific white matter tracts that affect recovery of various aspects of connected speech. The paper is well-written and most of the methods are sound.

The major weakness of the study is one that they identify themselves: “To maximize power to detect factors which affected recovery, we included the largest number of subjects tested both acutely and at a single subsequent time point. Although this approach helped avoid type II statistical errors, i.e. false negatives, it rendered direct comparisons between time points difficult to interpret.”

In fact, it makes all of the conclusions about the effect of lesions at one point on recovery at a later point impossible to interpret, because the patients are different patients. This would be a fatal flaw unless the authors re-analyzed their results using only the subset of patients who were tested at the same time points. Ideally, they would want to include only patients tested at ALL time points, especially for comparisons across the time points, like figures 3 & 4 and Table 1. These figures and tables do not make any sense, since each time point represented different subsets of patients. However, clearly that is not possible to do the ideal approach (and understandably so, as patients simply are unable to be tested at certain time points for various reasons). At the very least, they would need to make separate tables and figures that include the same subset of patients at each time point.

A few other suggestions:
1. Please report test-retest and inter-rater reliability for the connected speech analyses.
2. Although it is tempting to describe “statistically significant” results that do not survive correction for multiple comparisons, these results should at most be reported in the supplemental materials, as they could be due to chance alone.
3. You report, “Three patients were recruited with neurological signs of acute LH stroke but no clear lesion was identified from neuroimaging.” Why were these patients included? They were likely patients with stroke mimics, so they would not be appropriate for studying disruption of white matter tracts that affect connected speech (or its recovery). These patients should be removed, and the data re-analyzed.
4. Despite the limitations, there are some interesting findings such as, “During the subacute stage after stroke, acute FAT damage led to decreased recovery of fluency ($z = 2.79$, $p = 0.003$) and at the chronic stages (six – 12 months post-stroke) significantly decreased recovery of structural complexity (z values > 2.53, p values < 0.006) (see Figure 4).” However, the sentence would be much easier to read as separate sentences, especially since the 2 parts of the sentence are referring to different subsets of patients. For example, you could say, “Acute FAT damage led to decreased recovery of fluency at the subacute stage after stroke, ($z = 2.79$, $p = 0.003$). In an overlapping subset of patients, acute FAT

damage led to significantly decreased recovery of structural complexity at the chronic stages (six – 12 months post-stroke) (z values > 2.53, p values < 0.006).”
5. It is surprising that lesion volume did not predict the degree of connected speech recovery at any time point in the year after stroke even though initial severity did, since lesion volume and severity are generally highly correlated. Is this still true if you remove the 3 patients who had no visible lesions?

Very Minor:
p. 3 “…facilitate language intervention, it is important to identify factors which predict degree of connected speech recovery soon after stroke” should be changed to, “… facilitate language intervention, it is important to identify factors THAT predict degree of connected speech recovery soon after stroke.”
p.3 “Damage to disparate brain regions and their connections impairs word retrieval and its recovery but the neural predictors of the recovery of connected speech are unknown” requires a comma after recovery.
p.5 “It is unclear why damage to white matter tracts which connect different parts of the language network associated with different language functions have not been strongly differentiated with regards to connected speech,” should be changed to, “It is unclear why damage to white matter tracts THAT connect different parts of the language network associated with different language functions have not been strongly differentiated with regards to connected speech.”

Reviewer: 2

Comments to the Author
This is an interesting manuscript and is an important contribution to the literature given the multiple time-points assessed. However, I do have some concerns. My main concern is sample size. Although it initially sounds like you have a good number of participants, particularly for a longitudinal study, in Figure 5 where you highlight individuals in red who have damage to the FAT, this looks like a very small proportion of the participant total. This is highlighted again in your brain maps, where even the largest overlap in participants is just 4 people. This paper would benefit from more participants with damage to the FAT, in order to support your conclusions.

In the Introduction, although you do cite relevant literature, more detail could be added about papers which have found similar results - such as Alyahya et al., 2020 who also uses a lesion symptom mapping approach with stroke patients and highlighted the role of the FAT in the chronic stage. Although this manuscript builds on this with the addition of multiple timepoints, including the acute stage, better description of studies such as this in the Introduction would help the reader to understand your motivation for focusing on tracts such as the FAT.

On Figure 2, I understand that you are using performance within the normal range as a way to measure recovery, which is interesting. However, in this figure there are clearly individuals who are colored red (i.e., worse recovery) who do make large gains, albeit not to control range, and it concerns me that you are not addressing this. It would also be good to see comparisons between subacute-6mo, and 6mo-12mo, because it is difficult to see whether all the gains are in the acute-subacute stage, or if there are some individuals who have gains later into their recovery. The figure is also a little small, making it difficult to see individual trajectory.

On Figure 4, it is good to see the proportion of individuals with damage for each tract, but this would be much more informative if we could see the proportion of damage for each individual. If one participant only has damage to 10% of the tract, we might expect very different implications than for someone with 100% tract damage. You also don’t provide any information in the discussion regarding why fewer individuals have damage to these tracts at the later timepoints, which would be a good addition.

In Supplementary Table 1 you include correlations with different variables at each of the follow up timepoints, but it would be useful to see whether there are correlations with acute performance, particularly between acute performance and lesion volume.

Throughout the paper, it can be difficult to know which subset of patients you are referring to. For example on page 21, line 24, and page 22, line 8, you mention 80% of participants recovering, but does this include individuals who were initially in the control range? It would be good if you could be more specific throughout.

Finally, some of your methods could use more explanation for the motivation behind them. For example, why you chose to use results from a previous PCA rather than raw results, or why you chose to z-score the behavioral results rather than directly compare raw change, given that the same participants were used throughout. More detail of your motivation would help the reader to understand more about the methods, and why this might be a better/worse approach than other (more simple) options.

Overall, I think this is an excellent endeavor, and the results seem promising, particularly as they seem to align with results from the chronic stroke literature. However, I think the conclusions are a little strong, given the small proportion of individuals with damage to the regions of interest.

Comments from the Scientific Editor:

-Fig4: please report in the figure legend the test used and relevant test statistic values alongside p values (for example F or t values for ANOVAs and t-tests).

- Fig2 and Fig5: please, replace red colour with magenta to make the figure color blind friendly.

-Fig3-4: please, replace yellow colour with magenta to make the figure color blind friendly.

-Please include a paragraph about statistical analysis in the 'Materials and Methods' section.

-Please supply a graphical abstract. A graphical abstract is a visual representation of the central finding or methodology of the paper. It will be displayed in the table of contents. Authors should strive to make the graphical abstract informative, interesting, visually appealing, and straightforward (no caption needed, no abbreviations except gene names). Please upload as a separate high resolution image file (tiff preferred). The technical requirements for the graphical abstracts are:
--size of the submitted image: 1200 pixels square at 300dpi
--font of the text: Arial 12-16 point (smaller fonts will not be legible online)

-Please add an Abbreviated Summary (up to 50 words) of your paper that captures the main purpose and conclusions of the work. The summary should state the main results written in the third person ('[1st author surname] et al. report that …') and conclusion. This summary will be used in the contents list online and should not contain any abbreviations other than those on the accepted abbreviations list and accepted gene/protein names, making it accessible to the non-specialist. Please upload it as a Word document. Allowed abbreviations: AIDS; ANOVA; AMPA; ATP; A,T,C,G; CNS; CSF; CT; DNA; ECG; EEG; EMG; GABA; HIV; MRI; NMDA; PET; PCR; RNA.
