# Supplementary material for: Anterior connectivity critical for recovery of connected speech after stroke
Source: Brain Commun. 2022 Oct 19;4(6):fcac266. doi: 10.1093/braincomms/fcac266 (PMC9651028; doi:10.1093/braincomms/fcac266)
Supplement: fcac266_Supplementary_Data [file Revision 1 - Author's Response.pdf › Revision 1 - Author's Response.pdf]

Dear Scientific Editor,

We have formatted the manuscript based on the requirements.

Regarding Figure 4, this is a descriptive figure which visually demonstrates the proportion of patients out of the total cohort who did, or did not have damage to particular brain structures. We conducted NO statistical analyses related to these data. Thus, as we did not conduct any statistical tests (this is a descriptive data figure), we can report no relevant test statistic values (whether F or T or p values).

Sincerely yours
Tatiana Schnur and Junhua Ding
